# Efficacy and safety comparison of chemotherapies for advanced gastric cancer: A network meta-analysis

**DOI:** 10.18632/oncotarget.17784

**Published:** 2017-05-11

**Authors:** Jinping Sun, Zheng Ren, Xinfang Sun, Hongtao Hou, Ke Li, Quanxing Ge

**Affiliations:** ^1^ Department of Digestive Internal Medicine, Huaihe Hospital of Henan University, Kaifeng, 475000, Henan, China

**Keywords:** advanced gastric cancer, chemotherapies, network meta-analysis, efficacy, safety

## Abstract

**Objective:**

Chemotherapy is one of the commonly used therapies for advanced gastric cancer. In this study, we performed a network meta-analysis on the efficacy and safety of common treatments to give evidences of their relative benefits.

**Results:**

32 trials with 8550 patients and 20 regimens were included in this study. According to the results of primary outcomes, 5-FU plus OXA, 5-FU plus DOC, CAP plus CIS, CAP plus OXA, S-1 plus OXA and S-1 plus PAC performed well in improving OS and ORR. As for the adverse events, S-1 had a safer effect than other treatments, conversely, 5-FU plus CIS ranked the last. However, there was no regimen with outstanding performances in both efficacy and safety.

**Materials and Methods:**

Studies were searched from database and screened with criteria. The Bayesian framework based network meta-analysis was performed with software R and STATA. Overall survival (OS) and overall response rate (ORR) were considered as primary outcomes while adverse events as secondary outcomes. The outcomes were represented by hazard ratios or odd ratios with 95% corresponding credible intervals, respectively.

**Conclusions:**

The network meta-analysis suggested that 5-FU plus OXA and 5-FU plus DOC were recommended when efficacy was stressed. S-1 was safest but poorly effective. A regimen, as an excellent combination of efficacy and safety, is still waiting to be discovered.

## INTRODUCTION

Gastric cancer (GC) is a common cancer which leads to a second-high mortality around the world for several decades and caused the estimated 720,000 deaths in 2012 [1]. For the treatment of GC, resection operation has been considered as an optimal option for patients with early stage of GC. However, as the early symptoms of GC are nonspecific, it is hard to diagnose at an early stage and a majority of patients were diagnosed at phase II or III, known as unresectable AGC. So far, gold standard treatment for AGC has not been identified, and patients with AGC have a poor five-year survived rate, making it a challenging task in the clinical research.

Chemotherapy is one of the most common and effective therapies for AGC. A study conducted by Wagner *et al*. suggested that both overall survival (OS) and the life quality of patients treated with chemotherapy were significantly higher than patients with best supportive care [2]. Among all the chemotherapy drugs, fluorouracil (5-FU) and S-1 were the most conventional ones. 5-FU, which impacts on nucleoside metabolism and leads to cell death, has been used in GC patients since 1960s and its combined regimens have been suggested as effective therapeutic choice since 1980s [3]. 5-FU based combinations, such as 5-FU plus cisplatin (CIS), 5-FU plus adriamycin and mitomycin-C, 5-FU plus irinotecan (IRI), have been reported with an improvement of response rate from 10–15% (5-FU only) to 40–50% [4]. S-1, an oral combination of tegafur, gimeracil and oteracil, increases the concentration and half-life of 5-FU in plasma, and possibly circumvents its resistance [5]. Theoretically, S-1 based therapy can lead to a higher survival rate and fewer adverse events than 5-FU for patients with AGC. However, clinical practice seemed to contradict. For example, meta-analysis in 2016 suggested that S-1 had no difference in terms of OS and progression-free-survival (PFS) compared with 5-FU [6].

In addition to 5-FU and S-1, other drugs were also developed to treat AGC, including platinum drugs, such as CIS and oxaliplatin (OXA), and alcohol drugs, such as paclitaxel (PAC) and docetaxel (DOC), which were usually used in combination with S-1 and 5-FU. A meta-analysis conducted by Liu *et al*. reported that S-1 based combination therapies, including S-1 plus CIS, S-1 plus IRI and S-1 plus PAC was superior to S-1 were superior to S-1 monotherapy [7].

However, though many new drugs have been used in treating AGC, the progress of chemotherapy is not substantial in the past years mostly due to the drug resistance that tumor cells developed and sever adverse events chemotherapeutic created [8]; besides, the evaluation of existing regimens was insufficient because of the lack of reliable and comprehensive studies, although some efforts have been made. For example, a meta-analysis published in 2014 compared the difference between S-1-based therapy and non-S-1-based therapy and found that S-1-based therapy had no apparent advantage against capecitabine (CAP)-based therapy in efficacy for AGC [9]. Similarly, another meta-analysis in 2016 suggested that S-1 had no difference in terms of OS and progression-free-survival (PFS) compared with 5-FU [6].

Thus, aiming to draw a conclusion and provide reliable evidence, we designed a network meta-analysis with a large amount of data and compared the relative efficacy and safety of common chemotherapies in treating AGC. The relevant drugs include 5-FU, CAP, CIS, DOC, etoposide (ETO), IRI, lentinan (LNT), OXA, and PAC. Note that, because 5-FU is usually used in combination with leucovorin (LV, also named folinic acid) as the basis of most chemotherapy regimens for GC [10], here we regarded 5-FU intensified by leucovorin as an equivalent treatment to 5-FU alone as an adjustment for limited data.

## RESULTS

### Baseline characteristics of studies

**Figure 1:**
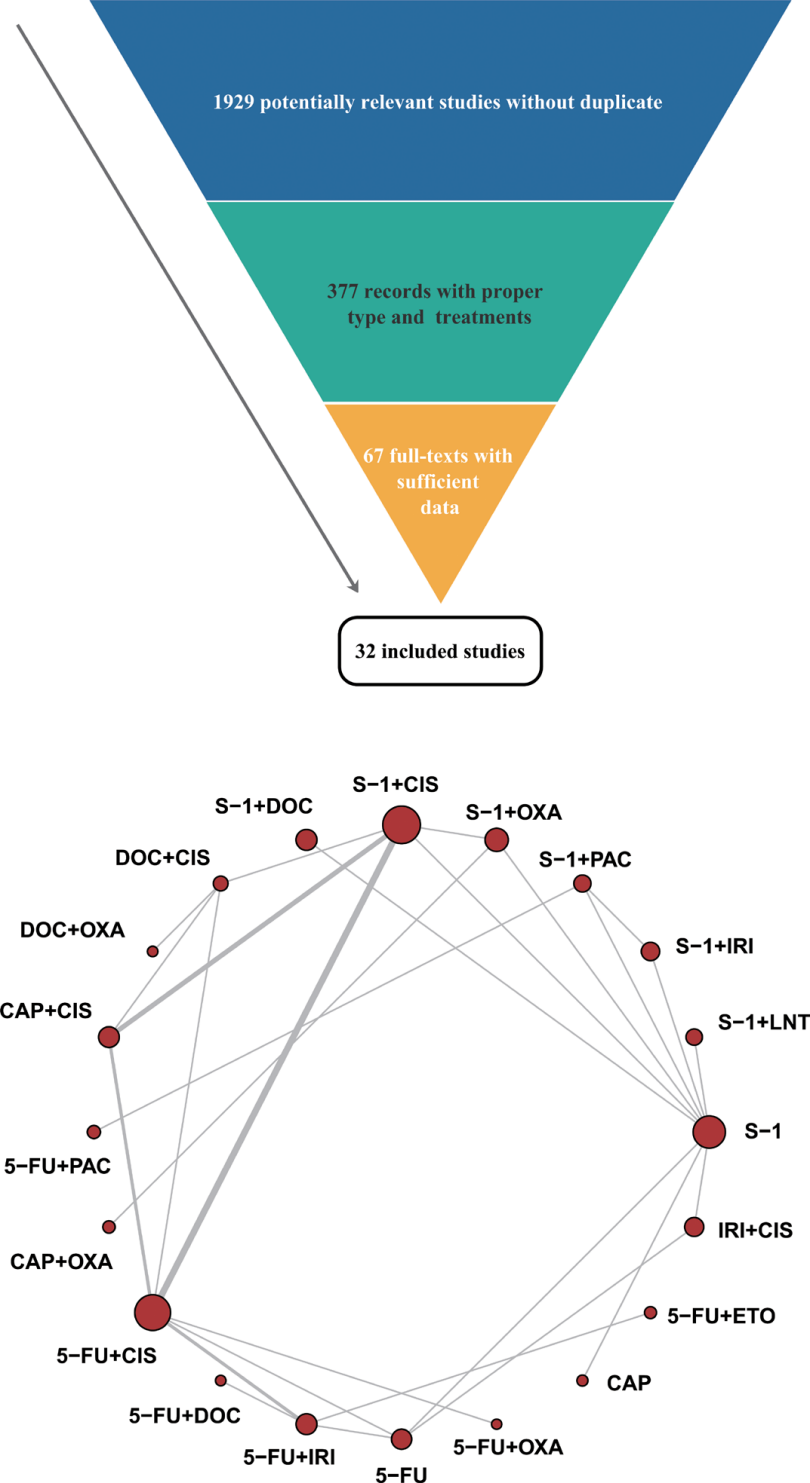
Flow chart and network structure for pain relief The network plots show direct comparison of different drugs, with node size corresponding to the sample size.

At first, 1,929 potentially relevant studies without duplicate were listed after literature search. After being screened with the inclusion criteria, 32 studies with 8,550 patients were finally included without disagreement [10–41]. The included studies, of which 28 were RCTs, 3 retrospective and 1 not specified, were all published between 2004 and 2016. 20 different therapeutic regimens with single or combined use of 10 drugs were contained. The literature searching process was showed in Figure 1. Other basic characteristics of the included studies were presented in Supplementary Table 1.

### Primary outcomes

#### 1-OS

**Figure 2:**
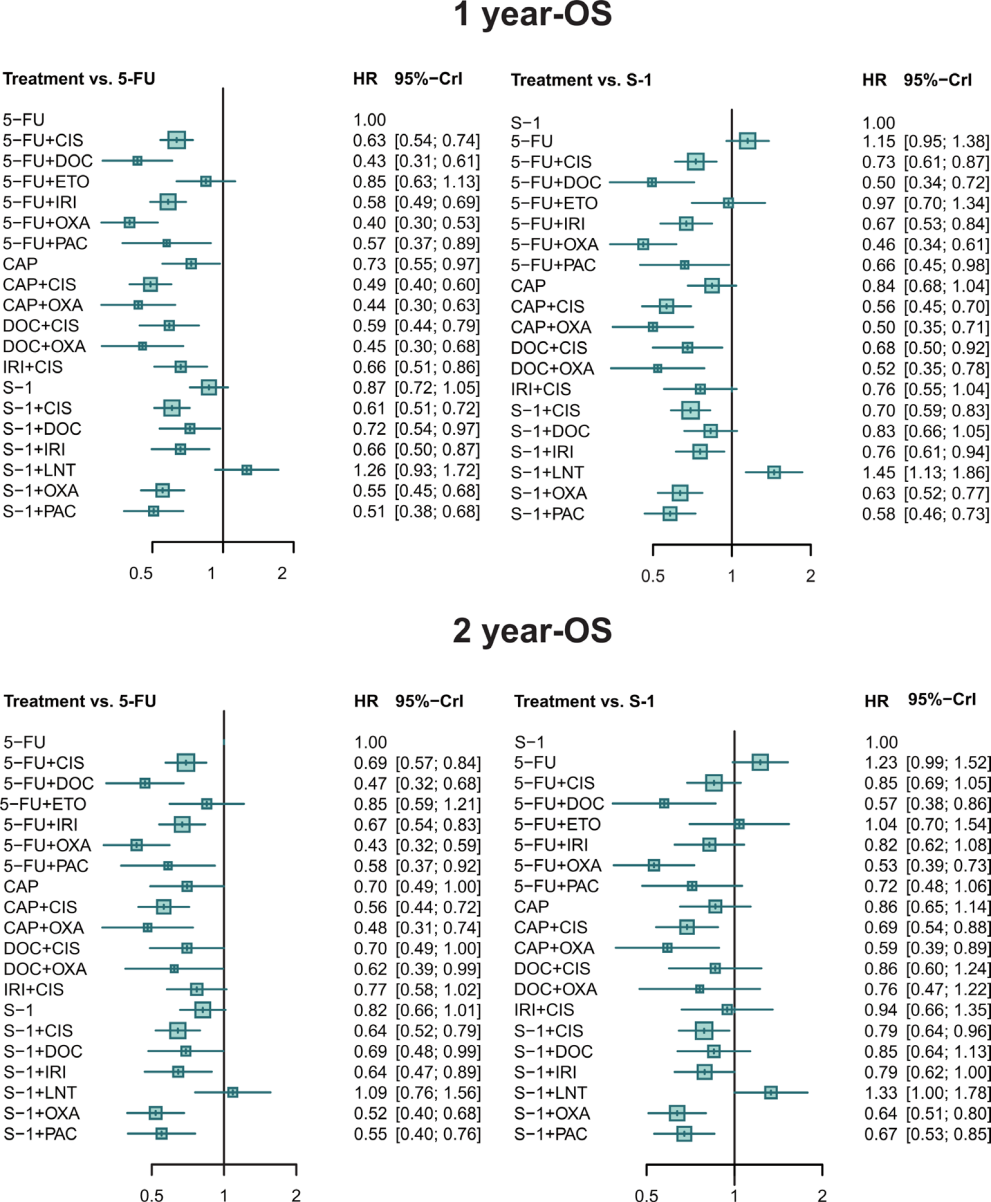
Forest plots for 1 year-OS and 2 year -OS Hazard ratios (HRs) with 95% credible interval (CrIs) indicate the relative efficacy.

According to the result of network meta-analysis (Figure 2 and Supplementary Table 2), 5-FU plus OXA showed superiority to 13 of the other 19 treatments. 5-FU plus DOC, CAP plus OXA, and CAP+CIS also exhibited desirable performance, significantly better than 10, 9, of the other treatments, respectively. Although not superior to most of the treatments, DOC plus OXA also yielded better results than 6 of the other treatments. .In the meantime, S-1 plus LNT turned out to be the worst choice, followed by 5-FU and S-1 monotherapy.

#### 2-OS

The result was similar to that of 1-OS (Figure 2 and Supplementary Table 2). 5-FU plus OXA was superior to most of the treatments while S-1 plus LNT was inferior to most of them followed by 5-FU and S-1. However, the performance of the combination of S-1 and OXA was relatively impressive, exhibiting better performance than 6 other treatments.

#### 3-OS

**Figure 3:**
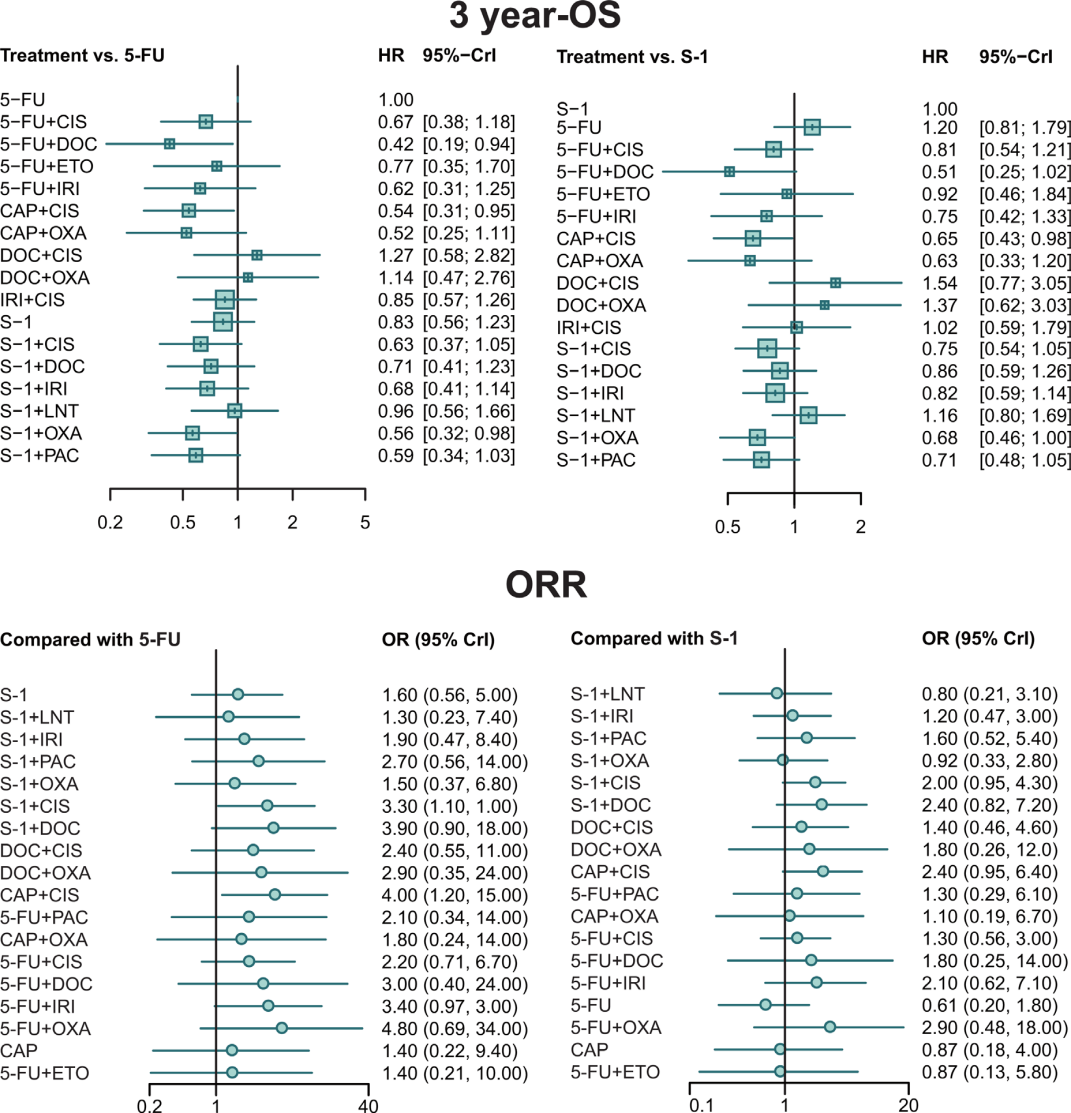
Forest plots for 3 year-OS and ORR Hazard ratios (HRs) and odds ratios (ORs) with 95% credible interval (CrIs) indicate the relative efficacy.

The results of 3-OS were presented in Supplementary Table 2 and Figure 3. For the absence of information with respect to 5-FU plus OXA5-FU plus DOC took its place to be the most effective treatment, yielding better results than S-1 plus LNT, 5-FU, 5-FU plus ETO, 5-FU plus IRI and DOC plus CIS. On the other hand, S-1 was significantly worse than S-1 plus OXA and CAP plus CIS.

### Overall response rate

As presented in Supplementary Table 2 and Figure 3, CAP plus CIS and S-1 plus CIS exhibited significantly better performance than 5-FU (OR = 4.01, 95% CrI = 1.15–14.73; OR = 3.35, 95% CrI = 1.05–10.80, respectively). However, no significant difference was detected among other treatments.

### Adverse events

With respect to anaemia and anorexia, S-1 performed better than S-1 plus CIS, CAP plus CIS and 5-FU plus CIS. 5-FU was associated with lower risk of diarrhea and fatigue compared to most of other treatments, while IRI plus CIS might lead to more febrile neutropenia and leucopenia events.S-1, S-1 plus IRI and CAP were associated with less nausea events, and S-1 and S-1 plus IRI were also effective in suppressing vomiting events. As for neutropenia, 5-FU, S-1 and IRI plus CIS were associated with lower risk. There was no difference among different treatments with respect to stomatitis and thrombocytopenia.

### Ranking of treatments

The results of SUCRA were presented in Table 1, showing the potential ranking probability of these treatments under each outcome. From the ranking of primary outcomes, S-1 plus PAC, CAP plus CIS, 5-FU plus DOC and 5-FU plus OXA were outstanding survival terms and ORR. Nevertheless, as lack of data in 3-OS with respect to 5-FU plus OXA, its efficacy on long-term survival was still uncertain. Additionally, although 5-FU plus DOC presented a high ranking at efficacy, its high risk in both anorexia and diarrhea and insufficient evidence in other adverse events made it seemingly not that reliable. In the case of adverse events, both S-1 and CAP exhibited relatively high ranking, which indicated that they were potentially the safest treatments. And with lowest ranking in most safety outcomes, 5-FU plus CIS should be greatly noted. It seemed that there was no ideal treatment which can perform well in both efficacy and safety.

**Table 1:** Surface under the cumulative ranking curve (SUCRA) results of OS, ORR and adverse events

Drug	1-OS	2-OS	3-OS	ORR	Anaemia	Anorexia	Diarrhoea	Fatigue	FN	Leu	Nausea	Neu	Stomatitis	Thr	Vomiting
**S-1**	0.134	0.197	0.320	0.329	0.795	0.870	0.435	0.712	0.770	0.676	0.850	0.802	0.649	0.696	0.767
**S-1+LNT**	0.005	0.036	0.216	0.281	0.604	0.672	0.198	0.298	0.843	0.520	0.252	0.347	0.347	0.276	0.497
**S-1+IRI**	0.398	0.513	0.544	0.422	0.765	0.710	0.608	0.514	0.624	0.605	0.836	0.365	0.365	0.638	0.875
**S-1+PAC**	0.743	0.737	0.698	0.591	0.342	0.536	0.612	0.551	0.825	0.707	0.440	0.502	0.502	0.767	0.385
**S-1+OXA**	0.640	0.800	0.738	0.313	0.602	0.338	0.443	0.491	0.662	0.538	0.355	0.585	0.585	0.292	0.619
**S-1+CIS**	0.502	0.527	0.626	0.702	0.242	0.215	0.384	0.335	0.403	0.239	0.280	0.647	0.647	0.273	0.366
**S-1+DOC**	0.298	0.421	0.505	0.746	-	0.732	0.602	0.592	0.276	0.245	0.684	0.386	0.386	-	0.639
**DOC+CIS**	0.536	0.399	0.099	0.516	-	-	-	-	-	-	-	-	-	-	-
**DOC+OXA**	0.825	0.567	0.187	0.588	-	-	-	-	-	-	-	-	-	-	-
**CAP+CIS**	0.784	0.719	0.794	0.780	0.188	0.318	0.616	0.055	0.568	0.348	0.303	0.611	0.611	0.352	0.323
**5-FU+PAC**	0.571	0.634	-	0.476	0.123	0.633	0.606	0.682	-	0.824	0.360	0.345	0.345	0.887	0.201
**CAP+OXA**	0.865	0.837	0.763	0.413	0.916	0.406	0.453	-	-	0.704	0.242	-	-	0.335	0.366
**5-FU+CIS**	0.419	0.397	0.538	0.461	0.293	0.272	0.599	0.280	0.219	0.217	0.139	0.247	0.247	0.270	0.223
**5-FU+DOC**	0.869	0.863	0.904	0.604	-	0.076	0.116	-	-	-	-	-	-	-	-
**5-FU+IRI**	0.561	0.467	0.623	0.697	0.521	0.453	0.185	-	0.397	0.350	0.423	0.513	0.513	0.658	0.371
**5-FU**	0.062	0.055	0.185	0.160	0.549	0.843	0.944	0.935	0.500	0.831	0.776	0.353	0.353	0.379	-
**5-FU+OXA**	0.934	0.934	-	0.764	-	0.518	0.726	0.689	-	-	-	-	-	-	-
**CAP**	0.284	0.405	-	0.325	0.816	0.867	0.272	-	0.563	0.627	0.951	0.599	0.599	0.634	0.885
**5-FU+ETO**	0.170	0.198	0.409	0.330	0.244	0.127	0.840	-	0.281	-	0.701	-	-	0.545	0.483
**IRI+CIS**	0.401	0.296	0.351	-	-	0.416	0.361	0.366	0.069	0.070	0.407	0.852	0.852	-	-

Note: FN = Febrile Neutropenia; Leu = Leucopenia; Neu = Neutropenia; Thr= Thrombocytopenia. The red color indicates good ranking, while green means bad result.

### Node-spitting results and net heat plots

**Figure 4:**
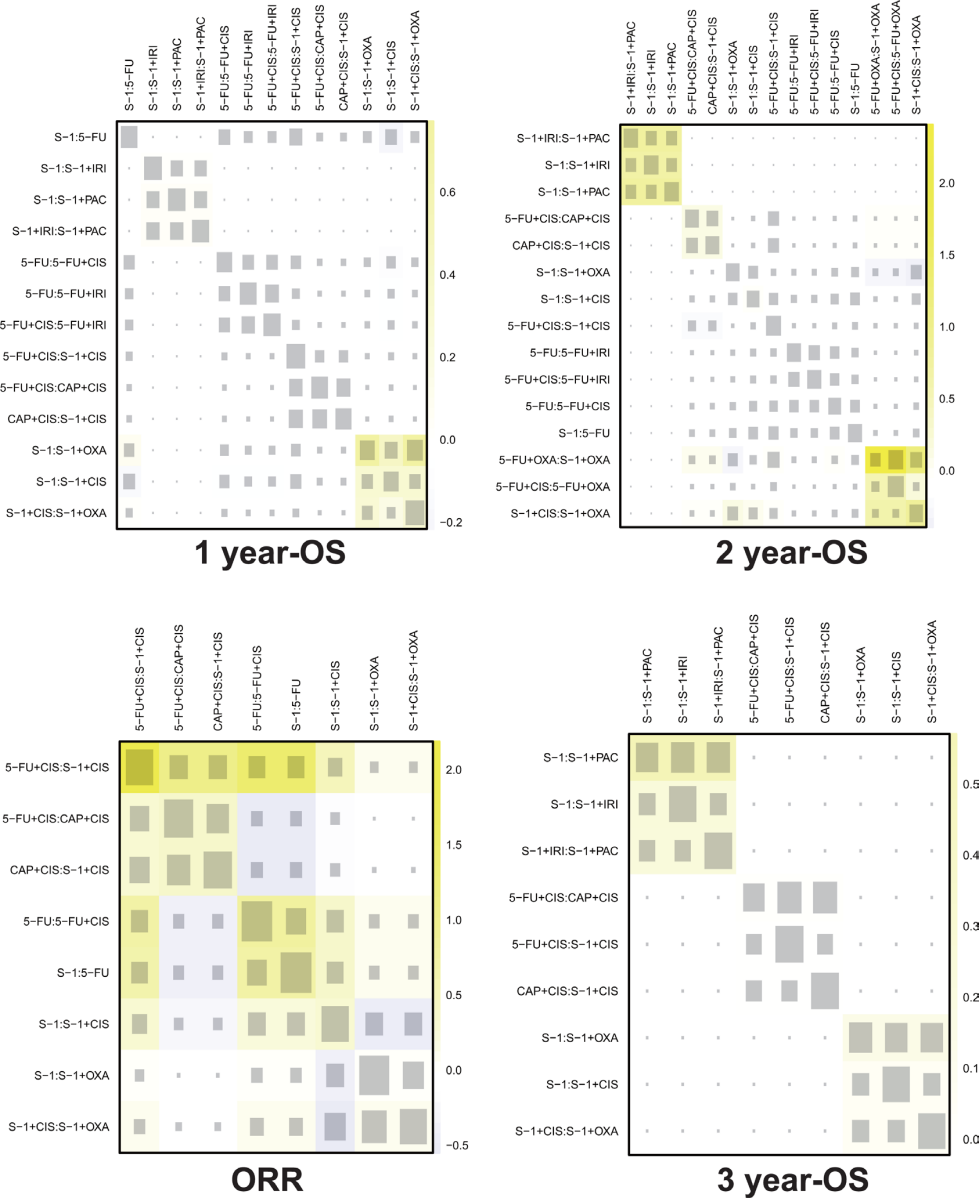
Heat plots for OS and ORR The size of the gray squares indicates the contribution of the direct evidence (shown in the column) to the network evidence (shown in the row). The colors are associated with the change in inconsistency between direct and indirect evidence (shown in the row). Blue colors indicate an increase of inconsistency and warm colors indicate a decrease.

To confirm the consistency between the corresponding results of direct comparison, indirect comparison and network analysis, the node-splitting analysis of outcomes with *P*-value was shown as Supplementary Table 3. A *P*-value < 0.05 indicates a significant inconsistency. According to the results, most of the network analyses were consistent with the direct comparison with a *P*-value lager than 0.05. However, there were several exceptions: when compared with S-1 plus OXA in ORR, the node-splitting results of S-1 and S-1 plus CIS showed significant inconsistency (*P*-value = 0.024 and 0.023, respectively). Besides, the comparison of S-1 versus S-1 plus IRI in terms of leucopenia was also indicated as significant inconsistency with a *P*-value of 0.034. In addition, the net heat plots of primary outcomes were showed as Figure 4. Similar with the result of node-spitting, the net heat plots illustrate little heterogeneity of the evidence.

## DISCUSSION

AGC keeps a high incidence and mortality around the world. Although many kinds of chemotherapies have been utilized for treating it, the OS is still poor because of the high probability of lurks and recurrence. To give valuable suggestions for treatments through comparing their efficacy and safety, we conducted the network meta-analysis among 20 chemotherapy regimens with 10 drugs commonly used for AGC, including S-1 based therapies, 5-FU based therapies and others.

The results demonstrated that 5-FU plus OXA may have a best efficacy in treating patients with AGC. OXA is a 3rd generation platinum compound with a better accumulation and safety profile in tumor cells than CIS. The mechanism of its antitumor action is suggested as its interference on DNA adducts formation by affecting DNA damage repairing proteins, transcription factors and DNA polymerases [42]. It has been verified that the combination chemotherapy of OXA and 5-FU is effective in 5-FU- and CIS-resistant cell lines, meanwhile 5-FU can increase the expression of *MRP2/ABCC2* pathway and induce a hypersensitivity to OXA [42, 43]. Results of this NMA supported its efficacy in improving OS and ORR; furthermore, it had a good performance in known adverse events including diarrhea and fatigue, which suggested that the combination regimen of 5-FU and OXA may have a great potential as a mainstream chemotherapy for AGC. However, due to the lack of evidence, most outcomes concerning safety under this treatment were not included in this study. Therefore, it should be further evaluated before any clinical commendation was given.

Similar to 5-FU plus OXA, the combination of 5-FU and DOC was outstanding for efficacy in both OS and ORR, but its risk in inducing anorexia and diarrhea was higher than most of the other treatments. DOC is one of the new chemotherapeutic drugs widely used for tumors since 1990s,and its promising antitumor activity in several cancers has been verified by previous studies, both as mono therapy and combined with other drugs [44]. It is also believed that the combination regimen DCF (DOC/CIS/5-FU) could become a standard therapy for advanced, recurrent and metastatic GC [44]. Nevertheless, along with its good efficacy, the high risk of toxicity, especially gastrointestinal toxicity, of double and triple DOC based chemotherapies were also indicated by Tetzlaff *et al*. [45]. According to our network meta-analysis, the incidence of anorexia and diarrhea among patients treated with 5-FU plus DOC was significantly higher than that of 5-FU mono therapy, which was in line with previous studies. Therefore, dosage must be administrated to control the adverse events in clinical use. To evaluate the efficacy and safety of 5-FU plus DOC for AGC accurately, more experimental data are needed.

Focusing on the adverse events, this NMA indicated that mono therapy of S-1 exhibited the best performance in preventing adverse event, while its efficacy was nearly the worst. On the other hand, S-1 based polytherapies, including S-1 plus PAC, S-1 plus OXA and S-1 plus CIS, exhibited better performance in efficacy, with an increasing risk of adverse events. In fact, there have already been several previous studies focusing on the difference between mono therapy and combination therapy of S-1: a meta-analysis conducted by Liu *et al*. analyzed four RCTs and indicated that S-1 based combination therapies led to a better OS, PFS and ORR than the mono therapy S-1 in treating AGC, but the mono therapy had a lower incidence of leucopenia, neutropenia and diarrhea, which was consistent with our results [46]. In fact, before this study, several previous network meta-analyses of chemotherapies for AGC have been published, however, most of them focused on the comparison of general types of regimens, such as S-1 based and 5-FU based, but not on certain treatments. For instance, a study published in 2016 suggested that both S-1 based and capecitabine based regimens had a significant improvement of OS when compared with 5-FU based regimens [47]. In this study, the data of specific therapies were analyzed to screen out the optimal regimens with relatively better efficacy and safety, which was a further attempt. The combination of direct and indirect comparison with a relatively large sample size and consistency added support for the reliability to our results.

However, several limitations of our analysis should also be taken into account. The node-splitting results and heat plots showed consistency between direct and indirect analysis of most of comparisons except that of S-1 plus OXA versus S-1 in ORR, S-1 plus OXA versus S-1 plus CIS in ORR, and S-1 versus S-1 plus IRI in leucopenia. A main source of the inconsistency was likely to be insufficiency of direct evidence. What's more, characteristics of included trials like sample size can make the indirect results quite different from the direct results in a way. To reduce the inconsistency and improve the reliability of the results, more pairwise comparison should be included in the further studies. Besides, results could be influenced by trial design as well. Several of our included trials were not randomized. And in some included trials, leucovorin was taken with 5-FU as an enhancer, the benefit solely brought by leucovorin was not separated or evaluated in this study. With a larger number of individual studies, the further analysis may get a more reliable result by narrowing the type of trials and classifying treatments more meticulously. Moreover, as the course of disease and dosage varied from patient to patient, the corresponding adjustment may contribute to a more accurate analysis.

In conclusion, our study demonstrated that 5-FU plus OXA and 5-FU plus DOC were the best two regimens for AGC when only considering efficacy, and S-1 was proved to be the safest one. However, the risk of 5-FU plus OXA or 5-FU plus DOC to cause adverse events still remained unclear, and S-1 had a relatively lower efficacy for treatment. A regimen, which has an excellent combination of efficacy and safety, still remains to be discovered.

## MATERIALS AND METHODS

### Literature search

We conducted a comprehensive search on databases including China National Knowledge Internet (CNKI), Embase and Pubmed for literature published between January 1st, 2000 and November 1st, 2016. Literature were identified with key words including “stomach neoplasms”, “chemotherapy”, “capecitabine”, “cisplatin”, “docetaxel”, “fluorouracil”, “irinotecan”, “lentinan”, “oxaliplatin”, “paclitaxel”, “etoposide”, “S-1” and their synonyms. Duplicate and irrelevant studies were excluded manually after being verified.

### Inclusion

To ensure the reliability of our outcomes, two investigators independently screened the title and abstract of the retrieved studies. Only studies fulfilling the following inclusion criteria were included: 1) diagnosis of AGC among all patients of the study should be confirmed; 2) data of patients, treatments and outcomes should be sufficient; 3) the study should be conducted with one or more pairwise comparison between the included treatments; 4) the outcomes of study should include at least one of the included primary outcomes or secondary outcomes.

### Outcomes

Primary outcomes included 1, 2, 3-year OS (1-OS, 2-OS, 3-OS) and overall response rate (ORR), which were used to measure the efficacy of different treatments. Secondary outcomes were adverse events (grade ≥ 3) including anemia, anorexia, diarrhea, fatigue, febrile neutropenia, leucopenia, nausea, neutropenia, stomatitis, thrombocytopenia and vomiting, which were measure the safety of different treatments.

### Data extraction

The data of included studies were extracted by two independent investigators. The baseline characteristics of all studies, including author, published year, study design, age and gender of patients, group size and treatments were collected. Besides, the data for outcomes as mentioned above were also extracted and recorded. Any discrepancy between the two investigators would be resolved by carrying a discussion to reach a consensual conclusion.

### Data analysis

Network meta-analysis was performed with Bayesian framework in software R (V3.3.1) and STATA (V13.0) based on our design. Primary outcomes including 1-OS, 2-OS and 3-OS were represented by hazard ratios (HRs) with 95% corresponding credible intervals (CrIs), while ORR and adverse events were represented by odds ratios (ORs) with 95% corresponding CrIs. Moreover, surface under the cumulative ranking curve (SUCRA) was calculated to show the potential ranking probability of each treatment under each outcome and to identify proper recommended treatments. In addition, node-splitting plots and net heat plots were computed to analyze the inconsistency between direct and indirect evidence.

## SUPPLEMENTARY TABLES








