# Supplementary material for: Efficacy and safety comparison of chemotherapies for advanced gastric cancer: A network meta-analysis
Source: Oncotarget. 2017 May 11;8(24):39673–82. doi: 10.18632/oncotarget.17784 (PMC5503642; doi:10.18632/oncotarget.17784)
Supplement: Supplementary file 1 [file oncotarget-08-39673-s001.pdf]

Efficacy and safety comparison of chemotherapies for advanced gastric cancer: A network meta-analysis

Supplementary Materials

Supplementary Table 1: Main characteristics of included studies. See Supplementary_Table_1

Supplementary Table 2: Network meta-analysis results for OS and ORR. See Supplementary_Table_2

Supplementary Table 3: Node-splitting results of the network meta-analysis for ORR and adverse events. See Supplementary_Table_3
