# Supplementary material for: Efficacy and safety comparison of chemotherapies for advanced gastric cancer: A network meta-analysis
Source: Oncotarget. 2017 May 11;8(24):39673–82. doi: 10.18632/oncotarget.17784 (PMC5503642; doi:10.18632/oncotarget.17784)
Supplement: Supplementary file 2 [file oncotarget-08-39673-s002.docx]

**Supplementary Table 1. Main characteristics of included studies**

| **ID** | **Study** | | | | |  | **Patients** | |  | **Treatments** | |
| --- | --- | --- | --- | --- | --- | --- | --- | --- | --- | --- | --- |
|  | **First Author** | **Year** | **Design** | **Blinding** | **Group Size** |  | **Median Age (Range)** | **Men (%)** |  | **Drug** | **Dosage** |
| 1 | Yoshino | 2016 | RCT | None | 146 |  | 74 (32-94) | 73.30 |  | S-1 | S-1: BSA |
|  |  |  |  |  | 149 |  | 73 (44-93) | 67.80 |  | S-1+LNT | S-1:BSA ; LNT: 2 mg/body |
| 2 | Sugimoto | 2014 | RCT | - | 50 |  | 64(25-75) | 76.00 |  | S-1+IRI | IRI:80 mg/m^2^ ; S-1:40 mg/m^2^ |
|  |  |  |  |  | 51 |  | 62(30-75) | 74.51 |  | S-1+PAC | PAC: 50 mg/m2,S-1:40 mg/m2 |
| 3 | Yamada | 2015 | RCT | None | 318 |  | 65(21-83) | 75.50 |  | S-1+OXA | S-1:80–120 mg/day ; OXA:100 mg/m^2^ |
|  |  |  |  |  | 324 |  | 65(29-85) | 73.10 |  | S-1+CIS | S-1:80–120 mg/day ; CIS:60 mg/m^2^ |
| 4 | Li | 2015 | RCT | - | 120 |  | 53(41-65) | 70.00 |  | S-1+CIS | S-1:40mg/m^2^ ; CIS:20mg/m^2^ |
|  |  |  |  |  | 116 |  | 55(44-66) | 73.30 |  | 5-FU+CIS | 5-FU:800 mg/m^2^ ; CIS:20mg/m^2^ |
| 5 | Lu | 2014 | RCT | - | 47 |  | 63(37-75) | 78.00 |  | S-1+OXA | S-1:BSA ; OXA:130 mg/m^2^ |
|  |  |  |  |  | 47 |  | 65(34-74) | 73.20 |  | S-1 | S-1:BSA |
| 6 | Koizumi | 2014 | RCT | None | 314 |  | 65(23-79) | 72.29 |  | S-1+DOC | S-1:80-120 mg BSA ; DOC:40 mg/m^2^ |
|  |  |  |  |  | 321 |  | 65(27-79) | 71.34 |  | S-1 | S-1:80-120 mg BSA |
| 7 | Kim | 2014 | RCT | None | 38 |  | 56(35-74) | 74.00 |  | DOC+CIS | DOC:35 mg/m^2^ ; CIS:60 mg/m^2^ on day 1 |
|  |  |  |  |  | 39 |  | 58(39-75) | 67.00 |  | DOC+OXA | DOC:35 mg/m^2^ ; OXA:120 mg/m^2^ on day 1 |
| 8 | Shitara | 2013 | Retro | - | 50 |  | 61(36-79) | 74.00 |  | S-1+CIS | S-1:80 mg/m^2^ ; CIS: 60 mg/m^2^ |
|  |  |  |  |  | 26 |  | 65(40-79) | 85.00 |  | CAP+CIS | CAP:1000 mg/m^2^ ; CIS:80 mg/m^2^ |
| 9 | Ajani | 2013 | RCT | None | 521 |  | 59(18-83) | 73.30 |  | S-1+CIS | S-1:25 mg/ m^2^ ; CIS:75 mg/m^2^ |
|  |  |  |  |  | 508 |  | 60(20-85) | 68.30 |  | 5-FU+CIS | 5-FU:1000 mg/m^2^/24 h ; CIS:100 mg/m^2^ |
| 10 | Nishikawa | 2012 | RCT | - | 38 |  | 67(48-79) | 65.80 |  | 5-FU+PAC(seq) | 5-FU:800 mg/m^2^ ; PAC:80 mg/m^2^ |
|  |  |  |  |  | 40 |  | 68(51-81) | 70.00 |  | S-1+PAC(seq) | S-1:80 mg/m^2^ ; PAC: 80 mg/m^2^ |
|  |  |  |  |  | 39 |  | 67(40-82) | 71.80 |  | 5-FU+PAC(con) | 5-FU:600 mg/m^2^ ; PAC:80 mg/m^2^ |
|  |  |  |  |  | 40 |  | 70(47-90) | 80.00 |  | S-1+PAC(con) | S-1:80 mg/m^2^ ; PAC:50 mg/m^2^ |
| 11 | Kim | 2012 | RCT | - | 65 |  | 60(28-77) | 68.00 |  | S-1+OXA | S-1: 80 mg/m^2^ ; OXA: 130 mg/m^2^ |
|  |  |  |  |  | 64 |  | 61(20-75) | 70.00 |  | CAP+OXA | CAP: 2000 mg/m^2^ ; OXA: 130 mg/m^2^ |
| 12 | Shen | 2011 | RCT | None | 80 |  | - | - |  | S-1 | S-1:80 mg/m^2^/d |
|  |  |  |  |  | 76 |  | - | - |  | S-1+CIS | S-1: 80mg/m^2^/d ; CIS:60mg/m^2^ |
|  |  |  |  |  | 74 |  | - | - |  | 5-FU+CIS | 5-FU:600mg/m^2^/d ; CIS: 20mg/m^2^/d |
| 13 | Narahara | 2011 | RCT | - | 162 |  | 63(27-75) | 79.00 |  | S-1 | S-1:80 mg/m^2^ |
|  |  |  |  |  | 164 |  | 63(33-75) | 71.00 |  | S-1+IRI | S-1:80 mg/m^2^ ; IRI:80 mg/m^2^ |
| 14 | Jeung | 2011 | RCT | - | 39 |  | 56(26-71) | 79.49 |  | S-1+DOC | S-1:35 mg/m^2^ ; DOC:35 mg/m^2^ |
|  |  |  |  |  | 41 |  | 60(22-75) | 68.29 |  | DOC+CIS | CIS:35 mg/m^2^ ; DOC:35 mg/m^2^ |
| 15 | Lim | 2010 | Retro |  | 77 |  | 59(35-77) | 71.43 |  | CAP+CIS | CAP:1000 mg/m^2^ bid po ; CIS:60-100 mg/m^2^ |
|  |  |  |  |  | 97 |  | 53(33-75) | 64.95 |  | S-1+CIS | S-1:40 mg/m^2^ bid po ; CIS:60-100 mg/m^2^ |
|  |  |  |  |  | 72 |  | 51(28-78) | 80.39 |  | DOC+CIS | DOC:75 mg/m^2^ iv ; CIS:60-100 mg/m^2^ |
|  |  |  |  |  | 37 |  | 60(30-75) | 54.05 |  | 5-FU+CIS | 5-FU:800-1000 mg/m^2^ iv ; CIS60-100 mg/m^2^ |
| 16 | Gubanski | 2010 | RCT | - | 39 |  | 63(39-79) | 66.67 |  | 5-FU*+DOC | DOC:45 mg/m^2^ ; 5-FU+Lv:bolus/44-h infusion |
|  |  |  |  |  | 39 |  | 64(42-75) | 87.18 |  | 5-FU*+IRI | IRI:180 mg/m^2^ ; 5-FU+Lv:bolus/44-h infusion |
| 17 | Ajani | 2010 | RCT | - | 521 |  | 59(18-83) | 73.30 |  | S-1+CIS | S-1: 50 mg/m^2^ ; CIS:75 mg/m^2^ |
|  |  |  |  |  | 508 |  | 60(20-85) | 26.70 |  | 5-FU+CIS | 5-FU:1,000 mg/m^2^/24 hours ; CIS:100 mg/m^2^ |
| 18 | Seol | 2009 | Retro |  | 32 |  | 73(70-85) | 84.38 |  | S-1+CIS | S-1:50 mg or 60 mg BSA ; CIS:70 mg/m^2^ |
|  |  |  |  |  | 40 |  | 74(70-82) | 80.00 |  | CAP+CIS | CAP:1250 mg/m^2^ ; CIS:70 mg/m^2^ |
| 19 | Sawaki | 2009 | RCT | - | 89 |  | 65 | - |  | 5-FU* | 5-FU:600 mg/m^2^ iv bolus ; I-LV:250mg/m^2^ 2h-iv |
|  |  |  |  |  | 88 |  | 63 | - |  | S-1 | S-1:40-60 mg BSA |
| 20 | Boku | 2009 | RCT | None | 234 |  | 63.5(57-69) | 75.21 |  | 5-FU | 5-FU:800 mg/m² per day |
|  |  |  |  |  | 236 |  | 63(59-68) | 76.27 |  | IRI+CIS | IRI:70 mg/m² ; CIS:80 mg/m² |
|  |  |  |  |  | 234 |  | 64(58-69) | 74.79 |  | S-1 | S-1:40 mg/m² |
| 21 | Popov | 2008 | - |  | 36 |  | 57(35-67) | 66.67 |  | 5-FU*+OXA | OXA:85 mg/m^2^ ; 5-FU:400 mg/m^2^ ; LV:200 mg/m^2^ |
|  |  |  |  |  | 36 |  | 55(31-69) | 72.22 |  | 5-FU*+CIS | CIS:50 mg/m^2^ ; 5-FU:400 mg/m^2^ ; LV: 200 mg/m^2^ |
| 22 | Lee | 2008 | RCT | None | 46 |  | 71(66-78) | 65.22 |  | CAP | 2500 mg/m^2^ |
|  |  |  |  |  | 45 |  | 71(65-82) | 82.22 |  | S-1 | 40–60 mg BSA |
| 23 | Koizumi | 2008 | RCT | None | 148 |  | 62(33-74) | 72.97 |  | S-1 | 40–60 mg BSA |
|  |  |  |  |  | 150 |  | 62(28-74) | 77.33 |  | S-1+CIS | S-1:40–60 mg BSA; CIS:60 mg/m² |
| 24 | Dank | 2008 | RCT | - | 170 |  | 58(29-76) | 73.53 |  | 5-FU*+IRI | 5-FU:2000 mg/m^2^; IRI:80 mg/m^2^ ; LV:500 mg/m^2^ |
|  |  |  |  |  | 163 |  | 59(28-77) | 66.26 |  | 5-FU+CIS | 5-FU:1000 mg/m^2^/day ; CIS:100 mg/m^2^ |
| 25 | Moehler | 2005 | RCT | None | 56 |  | 61(41-73) | 71.43 |  | 5-FU*+IRI | IRI:80 mg/m^2^ ; 5-FU:2000 mg/m^2^ ; LV:500 mg/m^2^ |
|  |  |  |  |  | 58 |  | 63(34-76) | 84.48 |  | 5-FU*+ETO | ETO:120 mg/m^2^ ; 5-FU:300 mg/m^2^ ; LV:500 mg/m^2^ |
| 26 | Bouche | 2004 | RCT | None | 45 |  | 64(45-75) | 82.00 |  | 5-FU* | LV:200 mg/m^2^ ; 5-FU:400 mg/m^2^ IV + 600 mg/m^2^ CI |
|  |  |  |  |  | 44 |  | 64(43-76) | 80.00 |  | 5-FU*+CIS | CIS:50 mg/m^2^ |
|  |  |  |  |  | 45 |  | 65(37-76) | 84.00 |  | 5-FU*+IRI | IRI:180 mg/m^2^ |
| 27 | Kobayashi | 2015 | RCT | - | 50 |  | 65 | 68.00 |  | S-1+CIS | S-1:40 mg/m^2^ bid ; CIS:60 mg/m^2^ |
|  |  |  |  |  | 50 |  | 65 | 68.00 |  | CAP+CIS | CAP:1000 mg/m^2^ bid ; CIS: 80 mg/m^2^ |
| 28 | Huang | 2013 | RCT | - | 119 |  | 56(18-74) | 74.79 |  | S-1+PAC | S-1:80–120 mg/d ; PAC:60 mg/m^2^ |
|  |  |  |  |  | 110 |  | 54(19-72) | 69.09 |  | 5-FU*+PAC | 5-FU:500 mg/m^2^ ; LV:20 mg/m^2^ ; PAC:60 mg/m^2^ |
| 29 | Wang | 2013 | RCT | None | 41 |  | 63(35-74) | 78.05 |  | S-1+PAC | S-1: BSA; PAC:60 mg/m^2^ |
|  |  |  |  |  | 41 |  | 61(31-73) | 73.17 |  | S-1 | S-1: BSA |
| 30 | Xu | 2013 | RCT | None | 120 |  |  |  |  | S-1+CIS | S-1:80 mg/m^2^/day, po ; CIS:20mg/m^2^ iv |
|  |  |  |  |  | 116 |  |  |  |  | 5-FU+CIS | 5-FU:0.8g/m^2^/d CI ; CIS:20mg/m^2^ iv |
| 31 | Kamatsu | 2011 | RCT | - | 48 |  | 70(47-78) | 70.83 |  | S-1+IRI | S-1:80−120mg/m^2^/day ; IRI:75 mg/m^2^ BSA |
|  |  |  |  |  | 47 |  | 63(24-76) | 78.72 |  | S-1 | S-1:80−120 mg/day |
| 32 | Kang | 2009 | RCT | None | 160 |  | 56(26-74) | 64.38 |  | CAP+CIS | CAP:1000 mg/m^2^; CIS:80 mg/m^2^ |
|  |  |  |  |  | 156 |  | 56(22-73) | 69.23 |  | 5-FU+CIS | 5-FU:800 mg/m^2^/day ; CIS:80 mg/m^2^ |

Note: Retro=retrospective research. 5-FU=Fluorouracil; CAP=Capecitabine; CIS=Cisplatin; DOC=Docetaxel; ETO=Etoposide; IRI=Irinotecan; LNT=Lentinan; OXA=Oxaliplatin; PAC=Paclitaxel. 5-FU* means 5-FU companied with leucovorin; BSA means “according to body's surface area”
