# Supplementary material for: Efficacy and safety comparison of chemotherapies for advanced gastric cancer: A network meta-analysis
Source: Oncotarget. 2017 May 11;8(24):39673–82. doi: 10.18632/oncotarget.17784 (PMC5503642; doi:10.18632/oncotarget.17784)
Supplement: Supplementary file 3 [file oncotarget-08-39673-s003.docx]

**Supplementary Table 2. Network meta-analysis results for OS and ORR**

|  |  | **S-1** | **S-1+LNT** | **S-1+PAC** | **S-1+IRI** | **S-1+OXA** | **S-1+CIS** | **5-FU+CIS** | **S-1+DOC** | **5-FU** | **5-FU+DOC** |  |
| --- | --- | --- | --- | --- | --- | --- | --- | --- | --- | --- | --- | --- |
| **1-OS** | **S-1** | **-** | **1.33 (1.00, 1.78)** | **0.67 (0.53, 0.85)** | **0.79 (0.62, 1.00)** | **0.64 (0.51, 0.80)** | **0.79 (0.64, 0.96)** | 0.85 (0.69, 1.05) | 0.85 (0.64, 1.13) | 1.23 (0.99, 1.52) | **0.57 (0.38, 0.86)** | **2-OS** |
|  | **S-1+LNT** | **1.45 (1.13, 1.86)** | **-** | **0.50 (0.35, 0.73)** | **0.59 (0.41, 0.86)** | **0.48 (0.33, 0.69)** | **0.59 (0.42, 0.84)** | **0.64 (0.45, 0.91)** | **0.64 (0.42, 0.96)** | 0.92 (0.64, 1.32) | **0.43 (0.26, 0.71)** |  |
|  | **S-1+PAC** | **0.58 (0.46, 0.73)** | **0.40 (0.29, 0.56)** | **-** | 1.17 (0.92, 1.49) | 0.95 (0.68, 1.31) | 1.17 (0.86, 1.59) | 1.26 (0.92, 1.74) | 1.26 (0.87, 1.83) | **1.82 (1.32, 2.51)** | 0.85 (0.53, 1.36) |  |
|  | **S-1+IRI** | **0.76 (0.61, 0.94)** | **0.52 (0.38, 0.72)** | 1.30 (1.04, 1.63) | **-** | 0.81 (0.58, 1.12) | 1.00 (0.73, 1.36) | 1.08 (0.78, 1.48) | 1.08 (0.74, 1.56) | **1.55 (1.12, 2.14)** | 0.73 (0.45, 1.16) |  |
|  | **S-1+OXA** | **0.63 (0.52, 0.77)** | **0.44 (0.32, 0.60)** | 1.09 (0.81, 1.47) | 0.84 (0.63, 1.12) | **-** | 1.24 (0.99, 1.54) | **1.34 (1.06, 1.69)** | 1.34 (0.93, 1.92) | **1.93 (1.47, 2.53)** | 0.90 (0.59, 1.38) |  |
|  | **S-1+CIS** | **0.70 (0.59, 0.83)** | **0.48 (0.35, 0.65)** | 1.20 (0.90, 1.59) | 0.92 (0.70, 1.21) | 1.10 (0.95, 1.26) | **-** | 1.08 (0.96, 1.22) | 1.08 (0.76, 1.53) | **1.56 (1.26, 1.93)** | 0.73 (0.50, 1.07) |  |
|  | **5-FU+CIS** | **0.73 (0.61, 0.87)** | **0.50 (0.37, 0.68)** | 1.25 (0.94, 1.67) | 0.96 (0.73, 1.27) | 1.15 (0.97, 1.36) | 1.05 (0.95, 1.15) | **-** | 1.00 (0.70, 1.43) | **1.44 (1.19, 1.75)** | **0.68 (0.47, 0.98)** |  |
|  | **S-1+DOC** | 0.83 (0.66, 1.05) | **0.57 (0.41, 0.80)** | **1.43 (1.04, 1.97)** | 1.10 (0.80, 1.50) | 1.31 (0.97, 1.77) | 1.19 (0.89, 1.59) | 1.14 (0.85, 1.53) | **-** | **1.44 (1.01, 2.07)** | 0.68 (0.41, 1.11) |  |
|  | **5-FU** | 1.15 (0.95, 1.38) | 0.79 (0.58, 1.08) | **1.98 (1.48, 2.64)** | **1.52 (1.14, 2.01)** | **1.81 (1.46, 2.24)** | **1.65 (1.39, 1.96)** | **1.58 (1.35, 1.85)** | **1.38 (1.03, 1.86)** | **-** | 0.47 (0.32, 0.68) |  |
|  | **5-FU+DOC** | **0.50 (0.34, 0.72)** | **0.34 (0.22, 0.53)** | 0.85 (0.55, 1.31) | **0.66 (0.43, 1.00)** | 0.78 (0.54, 1.13) | 0.71 (0.50, 1.01) | **0.68 (0.48, 0.96)** | **0.60 (0.39, 0.92)** | **0.43 (0.31, 0.61)** | **-** |  |

|  |  | **5-FU+ETO** | **5-FU+IRI** | **5-FU+OXA** | **5-FU+PAC** | **CAP** | **CAP+CIS** | **CAP+OXA** | **DOC+CIS** | **DOC+OXA** | **IRI+CIS** |  |
| --- | --- | --- | --- | --- | --- | --- | --- | --- | --- | --- | --- | --- |
| **1-OS** | **5-FU+ETO** | **-** | 0.79 (0.60, 1.04) | **0.51 (0.33, 0.79)** | 0.69 (0.40, 1.20) | 0.83 (0.51, 1.34) | **0.66 (0.45, 0.97)** | **0.57 (0.33, 0.97)** | 0.83 (0.52, 1.31) | 0.73 (0.42, 1.27) | 0.91 (0.58, 1.43) | **2-OS** |
|  | **5-FU+IRI** | **0.69 (0.55, 0.87)** | **-** | **0.65 (0.46, 0.90)** | 0.87 (0.54, 1.41) | 1.05 (0.71, 1.55) | 0.84 (0.64, 1.09) | 0.72 (0.45, 1.13) | 1.05 (0.72, 1.51) | 0.93 (0.57, 1.50) | 1.15 (0.80, 1.65) |  |
|  | **5-FU+OXA** | **0.47 (0.33, 0.68)** | **0.69 (0.52, 0.91)** | **-** | 1.35 (0.81, 2.24) | **1.62 (1.06, 2.47)** | 1.30 (0.96, 1.75) | 1.11 (0.70, 1.77) | **1.62 (1.10, 2.40)** | 1.43 (0.87, 2.36) | **1.78 (1.16, 2.73)** |  |
|  | **5-FU+PAC** | 0.68 (0.41, 1.13) | 0.99 (0.63, 1.55) | 1.44 (0.88, 2.34) | **-** | 1.20 (0.74, 1.94) | 0.96 (0.60, 1.53) | 0.82 (0.47, 1.45) | 1.20 (0.70, 2.05) | 1.06 (0.57, 1.97) | 1.32 (0.77, 2.24) |  |
|  | **CAP** | 0.87 (0.59, 1.27) | 1.25 (0.92, 1.71) | **1.83 (1.28, 2.62)** | 1.27 (0.82, 1.98) | **-** | 0.80 (0.55, 1.16) | 0.69 (0.42, 1.12) | 1.00 (0.63, 1.58) | 0.88 (0.51, 1.53) | 1.10 (0.70, 1.73) |  |
|  | **CAP+CIS** | **0.58 (0.42, 0.79)** | 0.84 (0.68, 1.04) | 1.23 (0.94, 1.60) | 0.85 (0.55, 1.33) | **0.67 (0.50, 0.91)** | **-** | 0.86 (0.56, 1.31) | 1.25 (0.89, 1.75) | 1.11 (0.70, 1.74) | 1.37 (0.94, 2.00) |  |
|  | **CAP+OXA** | **0.51 (0.33, 0.80)** | 0.75 (0.51, 1.08) | 1.09 (0.72, 1.64) | 0.76 (0.45, 1.28) | **0.59 (0.39, 0.90)** | 0.89 (0.62, 1.26) | **-** | 1.46 (0.88, 2.42) | 1.29 (0.71, 2.33) | 1.60 (0.95, 2.69) |  |
|  | **DOC+CIS** | 0.70 (0.48, 1.02) | 1.01 (0.75, 1.36) | **1.48 (1.06, 2.06)** | 1.03 (0.62, 1.68) | 0.81 (0.56, 1.17) | 1.20 (0.91, 1.59) | 1.36 (0.89, 2.06) | **-** | 0.88 (0.65, 1.20) | 1.10 (0.70, 1.73) |  |
|  | **DOC+OXA** | **0.54 (0.34, 0.85)** | 0.78 (0.52, 1.17) | 1.14 (0.74, 1.75) | 0.79 (0.45, 1.39) | **0.62 (0.39, 0.98)** | 0.93 (0.63, 1.37) | 1.04 (0.63, 1.72) | 0.77 (0.59, 1.01) | **-** | 1.24 (0.72, 2.15) |  |
|  | **IRI+CIS** | 0.78 (0.53, 1.15) | 1.13 (0.83, 1.55) | **1.65 (1.13, 2.41)** | 1.15 (0.69, 1.90) | 0.90 (0.62, 1.32) | 1.35 (0.97, 1.87) | 1.52 (0.97, 2.37) | 1.12 (0.76, 1.65) | 1.45 (0.90, 2.34) | **-** |  |

|  |  | **S-1** | **S-1+LNT** | **S-1+PAC** | **S-1+IRI** | **S-1+OXA** | **S-1+CIS** | **5-FU+CIS** | **S-1+DOC** | **5-FU** | **5-FU+DOC** |  |
| --- | --- | --- | --- | --- | --- | --- | --- | --- | --- | --- | --- | --- |
| **1-OS** | **5-FU+ETO** | 0.97 (0.70, 1.34) | **0.67 (0.45, 1.00)** | **1.67 (1.13, 2.47)** | 1.28 (0.87, 1.89) | **1.53 (1.11, 2.11)** | **1.40 (1.04, 1.88)** | **1.33 (1.00, 1.77)** | 1.17 (0.79, 1.74) | 0.85 (0.63, 1.13) | **1.96 (1.35, 2.84)** | **1-OS** |
|  | **5-FU+IRI** | **0.67 (0.53, 0.84)** | **0.46 (0.33, 0.65)** | 1.15 (0.84, 1.58) | 0.89 (0.65, 1.21) | 1.06 (0.84, 1.33) | 0.96 (0.79, 1.17) | 0.92 (0.77, 1.09) | 0.81 (0.58, 1.12) | **0.58 (0.49, 0.69)** | **1.35 (1.01, 1.81)** |  |
|  | **5-FU+OXA** | **0.46 (0.34, 0.61)** | **0.32 (0.22, 0.46)** | 0.79 (0.55, 1.14) | **0.61 (0.42, 0.87)** | **0.72 (0.55, 0.96)** | **0.66 (0.52, 0.84)** | **0.63 (0.50, 0.79)** | **0.55 (0.38, 0.80)** | **0.40 (0.30, 0.53)** | 0.93 (0.62, 1.39) |  |
|  | **5-FU+PAC** | **0.66 (0.45, 0.98)** | **0.46 (0.29, 0.72)** | 1.14 (0.82, 1.57) | 0.87 (0.59, 1.29) | 1.04 (0.67, 1.61) | 0.95 (0.62, 1.46) | 0.91 (0.59, 1.40) | 0.80 (0.51, 1.25) | **0.57 (0.37, 0.89)** | 1.33 (0.78, 2.28) |  |
|  | **CAP** | 0.84 (0.68, 1.04) | **0.58 (0.42, 0.80)** | **1.45 (1.06, 1.97)** | 1.11 (0.82, 1.50) | 1.32 (0.99, 1.77) | 1.21 (0.92, 1.59) | 1.15 (0.87, 1.53) | 1.01 (0.74, 1.39) | **0.73 (0.55, 0.97)** | **1.69 (1.11, 2.60)** |  |
|  | **CAP+CIS** | **0.56 (0.45, 0.70)** | **0.39 (0.28, 0.54)** | 0.97 (0.71, 1.32) | 0.74 (0.55, 1.01) | 0.89 (0.73, 1.08) | **0.81 (0.70, 0.93)** | **0.77 (0.67, 0.89)** | **0.68 (0.49, 0.93)** | **0.49 (0.40, 0.60)** | 1.14 (0.79, 1.64) |  |
|  | **CAP+OXA** | **0.50 (0.35, 0.71)** | **0.34 (0.22, 0.53)** | 0.86 (0.57, 1.31) | **0.66 (0.44, 1.00)** | 0.79 (0.59, 1.06) | **0.72 (0.52, 0.99)** | **0.69 (0.49, 0.96)** | **0.60 (0.39, 0.92)** | **0.44 (0.30, 0.63)** | 1.01 (0.63, 1.62) |  |
|  | **DOC+CIS** | **0.68 (0.50, 0.92)** | **0.47 (0.32, 0.69)** | 1.17 (0.80, 1.70) | 0.90 (0.62, 1.30) | 1.07 (0.79, 1.43) | 0.97 (0.75, 1.27) | 0.93 (0.73, 1.19) | 0.82 (0.56, 1.20) | **0.59 (0.44, 0.79)** | 1.37 (0.90, 2.08) |  |
|  | **DOC+OXA** | **0.52 (0.35, 0.78)** | **0.36 (0.22, 0.58)** | 0.90 (0.56, 1.43) | 0.69 (0.43, 1.09) | 0.82 (0.55, 1.23) | 0.75 (0.51, 1.09) | 0.72 (0.50, 1.03) | **0.63 (0.39, 1.00)** | **0.45 (0.30, 0.68)** | 1.05 (0.64, 1.73) |  |
|  | **IRI+CIS** | 0.76 (0.55, 1.04) | **0.52 (0.35, 0.78)** | 1.30 (0.88, 1.93) | 1.00 (0.68, 1.47) | 1.19 (0.85, 1.67) | 1.09 (0.80, 1.49) | 1.04 (0.77, 1.41) | 0.91 (0.62, 1.35) | **0.66 (0.51, 0.86)** | **1.53 (1.00, 2.35)** |  |

|  |  | **5-FU+ETO** | **5-FU+IRI** | **5-FU+OXA** | **5-FU+PAC** | **CAP** | **CAP+CIS** | **CAP+OXA** | **DOC+CIS** | **DOC+OXA** | **IRI+CIS** |  |
| --- | --- | --- | --- | --- | --- | --- | --- | --- | --- | --- | --- | --- |
| **2-OS** | **S-1** | 1.04 (0.70, 1.54) | 0.82 (0.62, 1.08) | **0.53 (0.39, 0.73)** | 0.72 (0.48, 1.06) | 0.86 (0.65, 1.14) | **0.69 (0.54, 0.88)** | **0.59 (0.39, 0.89)** | 0.86 (0.60, 1.24) | 0.76 (0.47, 1.22) | 0.94 (0.66, 1.35) | **2-OS** |
|  | **S-1+LNT** | 0.78 (0.48, 1.27) | **0.62 (0.41, 0.92)** | **0.40 (0.26, 0.61)** | **0.54 (0.33, 0.88)** | **0.65 (0.43, 0.96)** | **0.52 (0.35, 0.75)** | **0.44 (0.27, 0.73)** | 0.64 (0.40, 1.03) | **0.57 (0.33, 0.99)** | 0.71 (0.45, 1.12) |  |
|  | **S-1+PAC** | 1.54 (0.98, 2.44) | 1.22 (0.85, 1.75) | 0.79 (0.53, 1.17) | 1.06 (0.78, 1.46) | 1.28 (0.89, 1.84) | 1.02 (0.73, 1.44) | 0.88 (0.55, 1.40) | 1.28 (0.83, 1.97) | 1.13 (0.66, 1.92) | 1.40 (0.91, 2.16) |  |
|  | **S-1+IRI** | 1.31 (0.83, 2.08) | 1.04 (0.72, 1.49) | **0.67 (0.45, 1.00)** | 0.91 (0.61, 1.35) | 1.09 (0.76, 1.57) | 0.87 (0.62, 1.22) | 0.75 (0.47, 1.19) | 1.09 (0.70, 1.68) | 0.96 (0.57, 1.64) | 1.19 (0.78, 1.84) |  |
|  | **S-1+OXA** | **1.63 (1.08, 2.47)** | 1.29 (0.95, 1.75) | 0.83 (0.61, 1.14) | 1.13 (0.71, 1.77) | 1.35 (0.94, 1.93) | 1.08 (0.83, 1.41) | 0.93 (0.66, 1.30) | 1.35 (0.92, 1.97) | 1.19 (0.73, 1.94) | **1.48 (1.00, 2.20)** |  |
|  | **S-1+CIS** | 1.32 (0.91, 1.91) | 1.04 (0.82, 1.33) | **0.67 (0.51, 0.89)** | 0.91 (0.59, 1.41) | 1.09 (0.78, 1.54) | 0.87 (0.75, 1.02) | 0.75 (0.50, 1.12) | 1.09 (0.79, 1.50) | 0.97 (0.62, 1.50) | 1.20 (0.84, 1.71) |  |
|  | **5-FU+CIS** | 1.22 (0.86, 1.74) | 0.96 (0.78, 1.20) | **0.62 (0.48, 0.80)** | 0.84 (0.54, 1.32) | 1.01 (0.71, 1.43) | **0.81 (0.69, 0.95)** | 0.69 (0.46, 1.05) | 1.01 (0.75, 1.36) | 0.89 (0.58, 1.37) | 1.11 (0.79, 1.57) |  |
|  | **S-1+DOC** | 1.22 (0.75, 1.98) | 0.97 (0.65, 1.44) | **0.62 (0.41, 0.96)** | 0.84 (0.52, 1.37) | 1.01 (0.68, 1.51) | 0.81 (0.56, 1.18) | 0.69 (0.42, 1.14) | 1.01 (0.64, 1.61) | 0.89 (0.51, 1.56) | 1.11 (0.70, 1.76) |  |
|  | **5-FU** | 0.85 (0.59, 1.21) | **0.67 (0.54, 0.83)** | **0.43 (0.32, 0.59)** | **0.58 (0.37, 0.92)** | **0.70 (0.49, 1.00)** | **0.56 (0.44, 0.72)** | **0.48 (0.31, 0.74)** | **0.70 (0.49, 1.00)** | **0.62 (0.39, 0.99)** | 0.77 (0.58, 1.02) |  |
|  | **5-FU+DOC** | **1.81 (1.20, 2.72)** | **1.43 (1.06, 1.93)** | 0.92 (0.59, 1.44) | 1.25 (0.71, 2.19) | 1.50 (0.92, 2.45) | 1.20 (0.80, 1.79) | 1.03 (0.59, 1.77) | 1.50 (0.93, 2.40) | 1.32 (0.75, 2.33) | **1.64 (1.03, 2.62)** |  |

|  |  | **S-1** | **S-1+LNT** | **S-1+IRI** | **S-1+PAC** | **S-1+OXA** | **S-1+CIS** | **S-1+DOC** | **DOC+CIS** | **DOC+OXA** | **CAP+CIS** |  |
| --- | --- | --- | --- | --- | --- | --- | --- | --- | --- | --- | --- | --- |
| **3-OS** | **S-1** | **-** | 0.79 (0.21, 3.06) | 1.16 (0.47, 3.03) | 1.65 (0.52, 5.42) | 0.92 (0.33, 2.80) | 2.03 (0.95, 4.31) | 2.36 (0.82, 7.24) | 1.45 (0.46, 4.62) | 1.75 (0.26, 11.94) | 2.44 (0.95, 6.42) | **ORR** |
|  | **S-1+LNT** | 1.16 (0.80, 1.69) | **-** | 1.46 (0.29, 7.77) | 2.05 (0.35, 12.68) | 1.16 (0.22, 6.82) | 2.53 (0.55, 12.06) | 2.94 (0.54, 17.64) | 1.80 (0.31, 10.80) | 2.20 (0.22, 23.10) | 3.06 (0.60, 16.12) |  |
|  | **S-1+IRI** | 0.82 (0.59, 1.14) | 0.71 (0.43, 1.17) | **-** | 1.40 (0.44, 4.48) | 0.79 (0.20, 3.29) | 1.73 (0.51, 5.58) | 2.03 (0.49, 8.41) | 1.25 (0.28, 5.42) | 1.51 (0.18, 12.3) | 2.10 (0.55, 7.77) |  |
|  | **S-1+PAC** | 0.71 (0.48, 1.05) | 0.61 (0.36, 1.05) | 0.87 (0.63, 1.20) | **-** | 0.56 (0.12, 2.80) | 1.23 (0.30, 4.85) | 1.43 (0.29, 7.24) | 0.88 (0.17, 4.57) | 1.06 (0.11, 10.07) | 1.49 (0.32, 6.69) |  |
|  | **S-1+OXA** | **0.68 (0.46, 1.00)** | 0.59 (0.34, 1.01) | 0.83 (0.50, 1.39) | 0.96 (0.55, 1.67) | **-** | 2.20 (0.76, 5.81) | 2.56 (0.59, 10.70) | 1.57 (0.37, 6.23) | 1.90 (0.23, 14.73) | 2.66 (0.78, 8.41) |  |
|  | **S-1+CIS** | 0.75 (0.54, 1.05) | 0.65 (0.39, 1.07) | 0.92 (0.57, 1.47) | 1.06 (0.64, 1.78) | 1.11 (0.79, 1.55) | **-** | 1.16 (0.36, 3.97) | 0.71 (0.25, 2.05) | 0.86 (0.14, 5.58) | 1.21 (0.63, 2.36) |  |
|  | **S-1+DOC** | 0.86 (0.59, 1.26) | 0.74 (0.44, 1.26) | 1.05 (0.63, 1.74) | 1.21 (0.70, 2.09) | 1.26 (0.74, 2.17) | 1.14 (0.69, 1.89) | **-** | 0.61 (0.18, 1.99) | 0.74 (0.10, 5.16) | 1.04 (0.28, 3.71) |  |
|  | **DOC+CIS** | 1.54 (0.77, 3.05) | 1.32 (0.61, 2.90) | 1.87 (0.87, 4.02) | 2.16 (0.98, 4.78) | **2.26 (1.02, 4.97)** | 2.04 (0.95, 4.37) | **1.79 (1.01, 3.17)** | **-** | 1.21 (0.27, 5.58) | 1.68 (0.55, 5.21) |  |
|  | **DOC+OXA** | 1.37 (0.62, 3.03) | 1.18 (0.49, 2.84) | 1.67 (0.71, 3.95) | 1.93 (0.80, 4.68) | 2.01 (0.83, 4.87) | 1.82 (0.77, 4.29) | 1.59 (0.80, 3.20) | 0.89 (0.60, 1.32) | **-** | 1.39 (0.21, 9.21) |  |
|  | **CAP+CIS** | **0.65 (0.43, 0.98)** | **0.56 (0.32, 0.98)** | 0.79 (0.47, 1.35) | 0.92 (0.52, 1.62) | 0.95 (0.63, 1.44) | 0.86 (0.68, 1.09) | 0.76 (0.43, 1.32) | 0.42 (0.19, 0.94) | 0.47 (0.19, 1.16) | **-** |  |

|  |  | **S-1** | **S-1+LNT** | **S-1+IRI** | **S-1+PAC** | **S-1+OXA** | **S-1+CIS** | **S-1+DOC** | **DOC+CIS** | **DOC+OXA** | **CAP+CIS** |  |
| --- | --- | --- | --- | --- | --- | --- | --- | --- | --- | --- | --- | --- |
| **3-OS** | **5-FU+PAC** | - | - | - | - | - | - | - | - | - | - | **3-OS** |
|  | **CAP+OXA** | 0.63 (0.33, 1.20) | 0.54 (0.26, 1.14) | 0.77 (0.37, 1.58) | 0.89 (0.42, 1.88) | 0.93 (0.56, 1.54) | 0.84 (0.45, 1.54) | 0.73 (0.35, 1.54) | 0.41 (0.16, 1.05) | 0.46 (0.17, 1.27) | 0.97 (0.50, 1.87) |  |
|  | **5-FU+CIS** | 0.81 (0.54, 1.21) | 0.69 (0.40, 1.21) | 0.98 (0.58, 1.66) | 1.13 (0.64, 2.00) | 1.18 (0.79, 1.78) | 1.07 (0.85, 1.35) | 0.94 (0.54, 1.63) | 0.52 (0.24, 1.16) | 0.59 (0.24, 1.43) | 1.24 (0.93, 1.65) |  |
|  | **5-FU+DOC** | 0.51 (0.25, 1.02) | **0.44 (0.20, 0.97)** | 0.62 (0.29, 1.34) | 0.72 (0.32, 1.59) | 0.75 (0.37, 1.50) | 0.68 (0.37, 1.24) | 0.59 (0.27, 1.30) | 0.33 (0.12, 0.88) | 0.37 (0.13, 1.06) | 0.78 (0.42, 1.47) |  |
|  | **5-FU+IRI** | 0.75 (0.42, 1.33) | 0.65 (0.32, 1.28) | 0.91 (0.47, 1.78) | 1.06 (0.53, 2.12) | 1.10 (0.62, 1.96) | 0.99 (0.62, 1.59) | 0.87 (0.44, 1.73) | 0.49 (0.20, 1.20) | 0.55 (0.21, 1.45) | 1.15 (0.70, 1.90) |  |
|  | **5-FU** | 1.20 (0.81, 1.79) | 1.04 (0.60, 1.79) | 1.47 (0.88, 2.47) | 1.70 (0.97, 2.96) | **1.77 (1.02, 3.08)** | 1.60 (0.95, 2.67) | 1.40 (0.81, 2.42) | 0.78 (0.36, 1.73) | 0.88 (0.36, 2.13) | 1.85 (1.05, 3.27) |  |
|  | **5-FU+OXA** | - | - | - | - | - | - | - | - | - | - |  |
|  | **CAP** | - | - | - | - | - | - | - | - | - | - |  |
|  | **5-FU+ETO** | 0.92 (0.46, 1.84) | 0.80 (0.36, 1.75) | 1.13 (0.53, 2.43) | 1.30 (0.59, 2.88) | 1.36 (0.68, 2.71) | 1.23 (0.67, 2.24) | 1.08 (0.49, 2.36) | 0.60 (0.23, 1.59) | 0.67 (0.24, 1.93) | 1.42 (0.76, 2.66) |  |
|  | **IRI+CIS** | 1.02 (0.59, 1.79) | 0.88 (0.45, 1.73) | 1.25 (0.65, 2.40) | 1.44 (0.73, 2.86) | 1.50 (0.76, 2.97) | 1.36 (0.71, 2.60) | 1.19 (0.61, 2.34) | 0.67 (0.28, 1.62) | 0.75 (0.28, 1.97) | 1.58 (0.79, 3.15) |  |

|  |  | **5-FU+PAC** | **CAP+OXA** | **5-FU+CIS** | **5-FU+DOC** | **5-FU+IRI** | **5-FU** | **5-FU+OXA** | **CAP** | **5-FU+ETO** | **IRI+CIS** |  |
| --- | --- | --- | --- | --- | --- | --- | --- | --- | --- | --- | --- | --- |
| **ORR** | **S-1** | 1.30 (0.29, 6.05) | 1.08 (0.19, 6.69) | 1.31 (0.56, 3.00) | 1.84 (0.25, 13.60) | 2.10 (0.63, 7.03) | 0.61 (0.20, 1.80) | 2.89 (0.48, 17.64) | 0.87 (0.18, 4.01) | 0.87 (0.13, 5.81) | - | **ORR** |
|  | **S-1+LNT** | 1.63 (0.22, 12.81) | 1.36 (0.15, 13.33) | 1.63 (0.33, 7.92) | 2.32 (0.21, 25.79) | 2.64 (0.43, 15.96) | 0.76 (0.13, 4.26) | 3.63 (0.39, 34.47) | 1.08 (0.14, 8.41) | 1.09 (0.10, 11.13) | - |  |
|  | **S-1+IRI** | 1.12 (0.25, 4.95) | 0.93 (0.13, 6.96) | 1.13 (0.31, 3.82) | 1.58 (0.17, 14.15) | 1.79 (0.38, 8.08) | 0.52 (0.12, 2.12) | 2.48 (0.32, 18.73) | 0.75 (0.12, 4.35) | 0.75 (0.09, 6.11) | - |  |
|  | **S-1+PAC** | 0.79 (0.30, 2.05) | 0.66 (0.08, 5.64) | 0.79 (0.18, 3.29) | 1.12 (0.11, 11.13) | 1.27 (0.23, 6.75) | 0.37 (0.07, 1.79) | 1.77 (0.20, 14.88) | 0.53 (0.07, 3.53) | 0.53 (0.06, 4.90) | - |  |
|  | **S-1+OXA** | 1.40 (0.22, 8.76) | 1.17 (0.28, 4.85) | 1.42 (0.42, 4.22) | 1.99 (0.23, 16.61) | 2.27 (0.50, 9.30) | 0.66 (0.15, 2.66) | 3.13 (0.43, 21.54) | 0.94 (0.14, 5.81) | 0.94 (0.11, 7.10) | - |  |
|  | **S-1+CIS** | 0.64 (0.12, 3.63) | 0.53 (0.10, 3.19) | 0.64 (0.35, 1.17) | 0.90 (0.13, 6.36) | 1.03 (0.34, 3.13) | **0.30 (0.09, 0.95)** | 1.43 (0.26, 7.92) | 0.43 (0.08, 2.34) | 0.43 (0.07, 2.72) | - |  |
|  | **S-1+DOC** | 0.55 (0.08, 3.53) | 0.46 (0.06, 3.60) | 0.55 (0.15, 1.88) | 0.78 (0.09, 6.69) | 0.89 (0.19, 4.01) | 0.26 (0.06, 1.11) | 1.22 (0.16, 9.12) | 0.37 (0.05, 2.32) | 0.37 (0.04, 3.03) | - |  |
|  | **DOC+CIS** | 0.90 (0.13, 6.23) | 0.75 (0.10, 5.70) | 0.90 (0.30, 2.69) | 1.27 (0.15, 10.70) | 1.45 (0.34, 6.05) | 0.42 (0.09, 1.80) | 2.01 (0.29, 13.87) | 0.60 (0.09, 4.01) | 0.61 (0.08, 4.76) | - |  |
|  | **DOC+OXA** | 0.75 (0.07, 8.58) | 0.62 (0.05, 8.08) | 0.75 (0.11, 4.81) | 1.05 (0.08, 14.44) | 1.20 (0.15, 9.58) | 0.35 (0.04, 2.89) | 1.65 (0.14, 19.30) | 0.49 (0.04, 5.75) | 0.50 (0.04, 6.30) | - |  |
|  | **CAP+CIS** | 0.53 (0.09, 3.29) | 0.44 (0.07, 2.92) | 0.54 (0.25, 1.11) | 0.75 (0.10, 5.42) | 0.85 (0.25, 2.80) | **0.25 (0.07, 0.87)** | 1.19 (0.20, 6.89) | 0.35 (0.06, 2.12) | 0.36 (0.05, 2.34) | - |  |

|  |  | **5-FU+PAC** | **CAP+OXA** | **5-FU+CIS** | **5-FU+DOC** | **5-FU+IRI** | **5-FU** | **5-FU+OXA** | **CAP** | **5-FU+ETO** | **IRI+CIS** |  |
| --- | --- | --- | --- | --- | --- | --- | --- | --- | --- | --- | --- | --- |
| **3-OS** | **5-FU+PAC** | **-** | 0.84 (0.08, 8.76) | 1.01 (0.17, 5.58) | 1.42 (0.11, 17.12) | 1.6 (0.23, 11.02) | 0.47 (0.07, 2.92) | 2.23 (0.21, 23.1) | 0.67 (0.08, 5.58) | 0.67 (0.06, 7.39) | - | **ORR** |
|  | **CAP+OXA** | - | **-** | 1.21 (0.19, 7.17) | 1.68 (0.13, 21.98) | 1.93 (0.25, 14.44) | 0.56 (0.07, 4.14) | 2.66 (0.23, 29.37) | 0.79 (0.07, 8.00) | 0.80 (0.06, 9.58) | - |  |
|  | **5-FU+CIS** | - | 1.28 (0.67, 2.46) | **-** | 1.40 (0.23, 9.12) | 1.60 (0.61, 4.26) | 0.46 (0.15, 1.42) | 2.20 (0.45, 11.13) | 0.66 (0.12, 3.82) | 0.66 (0.11, 3.90) | - |  |
|  | **5-FU+DOC** | - | 0.81 (0.34, 1.91) | 0.63 (0.36, 1.11) | **-** | 1.14 (0.24, 5.37) | 0.33 (0.04, 2.48) | 1.58 (0.14, 17.64) | 0.47 (0.04, 5.70) | 0.48 (0.05, 4.06) | - |  |
|  | **5-FU+IRI** | - | 1.19 (0.55, 2.57) | 0.93 (0.62, 1.40) | **1.47 (1.00, 2.17)** | **-** | 0.29 (0.08, 1.03) | 1.38 (0.21, 8.94) | 0.41 (0.06, 2.89) | 0.42 (0.09, 1.80) | - |  |
|  | **5-FU** | - | 1.91 (0.90, 4.06) | 1.50 (0.85, 2.63) | 2.37 (1.06, 5.25) | 1.61 (0.80, 3.23) | **-** | 4.76 (0.68, 34.47) | 1.42 (0.22, 9.39) | 1.43 (0.21, 10.18) | - |  |
|  | **5-FU+OXA** | - | - | - | - | - | - | **-** | 0.30 (0.03, 3.13) | 0.30 (0.03, 3.16) | - |  |
|  | **CAP** | - | - | - | - | - | - | - | **-** | 1.00 (0.09, 11.59) | - |  |
|  | **5-FU+ETO** | - | 1.47 (0.62, 3.46) | 1.15 (0.66, 2.00) | **1.82 (1.06, 3.12)** | 1.23 (0.85, 1.80) | 0.77 (0.35, 1.70) | - | - | **-** | - |  |
|  | **IRI+CIS** | - | 1.63 (0.69, 3.80) | 1.27 (0.64, 2.53) | 2.01 (0.83, 4.90) | 1.37 (0.61, 3.05) | 0.85 (0.57, 1.26) | - | - | 1.11 (0.46, 2.69) | - |  |

Note: the **bold** form means significant result.
