# Supplementary material for: Efficacy and safety comparison of chemotherapies for advanced gastric cancer: A network meta-analysis
Source: Oncotarget. 2017 May 11;8(24):39673–82. doi: 10.18632/oncotarget.17784 (PMC5503642; doi:10.18632/oncotarget.17784)
Supplement: Supplementary file 4 [file oncotarget-08-39673-s004.docx]

**Supplementary Table 3. Node-splitting results of the network meta-analysis for ORR and adverse events**

| **Outcome** | **Comparison** | | | **Odds Ratio (95%CrI)** | | | ***p*-value** |
| --- | --- | --- | --- | --- | --- | --- | --- |
|  |  |  |  | **Direct** | **Indirect** | **Network** | **(Direct vs. Indirect)** |
| **ORR** | **S-1+IRI** | vs | **S-1** | 0.95 (0.36, 2.60) | 2.90 (0.36, 27.00) | 1.20 (0.49, 3.10) | 0.351 |
|  | **S-1+PAC** | vs | **S-1** | 2.90 (0.56, 15.00) | 0.81 (0.15, 5.00) | 1.70 (0.55, 5.00) | 0.269 |
|  | **S-1+OXA** | vs | **S-1** | 2.70 (0.81, 9.40) | 0.31 (0.09, 1.20) | 0.94 (0.36, 2.80) | **0.024** |
|  | **S-1+CIS** | vs | **S-1** | 1.70 (0.64, 4.40) | 3.40 (0.90, 11.00) | 2.00 (1.00, 4.10) | 0.390 |
|  | **S-1+DOC** | vs | **S-1** | 1.80 (0.48, 6.20) | 6.00 (0.82, 44.00) | 2.30 (0.81, 6.70) | 0.286 |
|  | **5-FU+CIS** | vs | **S-1** | 0.67 (0.17, 3.20) | 1.70 (0.57, 5.10) | 1.30 (0.58, 2.80) | 0.311 |
|  | **5-FU** | vs | **S-1** | 0.75 (0.18, 3.00) | 0.44 (0.06, 2.50) | 0.59 (0.21, 1.70) | 0.615 |
|  | **S-1+PAC** | vs | **S-1+IRI** | 0.89 (0.20, 3.70) | 3.10 (0.43, 21.00) | 1.40 (0.45, 4.20) | 0.285 |
|  | **S-1+CIS** | vs | **S-1+OXA** | 4.80 (1.60, 13.00) | 0.53 (0.14, 2.10) | 2.20 (0.78, 5.50) | **0.023** |
|  | **DOC+CIS** | vs | **S-1+CIS** | 0.75 (0.20, 3.10) | 0.27 (0.04, 2.60) | 0.73 (0.26, 2.00) | 0.411 |
|  | **CAP+CIS** | vs | **S-1+CIS** | 1.20 (0.54, 2.70) | 1.10 (0.21, 5.10) | 1.20 (0.64, 2.40) | 0.935 |
|  | **5-FU+CIS** | vs | **S-1+CIS** | 0.64 (0.29, 1.40) | 0.69 (0.16, 3.10) | 0.65 (0.34, 1.10) | 0.944 |
|  | **DOC+CIS** | vs | **S-1+DOC** | 0.37 (0.08, 1.60) | 1.30 (0.19, 7.70) | 0.62 (0.19, 2.00) | 0.331 |
|  | **CAP+CIS** | vs | **DOC+CIS** | 1.20 (0.28, 5.20) | 4.90 (0.44, 51.00) | 1.70 (0.59, 5.00) | 0.291 |
|  | **5-FU+CIS** | vs | **DOC+CIS** | 0.55 (0.12, 2.50) | 2.40 (0.23, 27.00) | 0.89 (0.29, 2.50) | 0.278 |
|  | **5-FU+CIS** | vs | **CAP+CIS** | 0.50 (0.20, 1.40) | 0.50 (0.13, 1.50) | 0.53 (0.25, 1.10) | 0.991 |
|  | **5-FU** | vs | **5-FU+CIS** | 0.38 (0.06, 2.30) | 0.61 (0.10, 3.70) | 0.46 (0.15, 1.30) | 0.730 |
|  | **5-FU** | vs | **5-FU+IRI** | 0.22 (0.04, 1.30) | 0.46 (0.05, 4.90) | 0.28 (0.08, 1.00) | 0.590 |
| **Anaemia** | **S-1+IRI** | vs | **S-1** | 1.30 (0.63, 2.60) | 6.70 (0.83, 59.00) | 1.50 (0.80, 3.20) | 0.139 |
|  | **S-1+PAC** | vs | **S-1** | 5.20 (1.30, 28.00) | 1.00 (0.21, 5.40) | 2.40 (0.85, 7.90) | 0.140 |
|  | **S-1+OXA** | vs | **S-1** | 2.30 (0.32, 20.00) | 5.70 (1.50, 23.00) | 4.70 (1.50, 13.00) | 0.488 |
|  | **S-1+CIS** | vs | **S-1** | 7.00 (2.60, 22.00) | 2.50 (0.33, 27.00) | 6.10 (2.50, 15.00) | 0.385 |
|  | **S-1+PAC** | vs | **S-1+IRI** | 0.79 (0.17, 3.40) | 4.20 (0.83, 27.00) | 1.60 (0.57, 4.90) | 0.135 |
|  | **S-1+CIS** | vs | **S-1+OXA** | 1.20 (0.49, 2.70) | 3.20 (0.30, 25.00) | 1.30 (0.64, 3.20) | 0.420 |
|  | **CAP+CIS** | vs | **S-1+CIS** | 0.69 (0.29, 1.50) | 3.50 (0.37, 96.00) | 0.81 (0.36, 1.70) | 0.169 |
|  | **5-FU+CIS** | vs | **S-1+CIS** | 0.98 (0.56, 1.70) | 0.17 (0.01, 2.20) | 0.89 (0.52, 1.50) | 0.201 |
|  | **5-FU+CIS** | vs | **CAP+CIS** | 0.25 (0.01, 2.60) | 1.40 (0.55, 4.00) | 1.10 (0.46, 2.90) | 0.223 |
| **Anorexia** | **S-1+IRI** | vs | **S-1** | 0.43 (0.13, 2.10) | 12.00 (0.21, 1500.00) | 0.59 (0.19, 2.70) | 0.128 |
|  | **S-1+PAC** | vs | **S-1** | 2.30 (0.14, 120.00) | 0.11 (0.00, 2.20) | 0.53 (0.06, 4.40) | 0.129 |
|  | **S-1+OXA** | vs | **S-1** | 2.40 (0.12, 100.00) | 0.75 (0.09, 5.40) | 1.00 (0.23, 5.30) | 0.516 |
|  | **S-1+CIS** | vs | **S-1** | 1.40 (0.39, 6.00) | 0.82 (0.05, 13.00) | 1.10 (0.34, 3.60) | 0.723 |
|  | **5-FU+CIS** | vs | **S-1** | 0.20 (0.00, 2.60) | 0.74 (0.11, 3.90) | 0.62 (0.14, 1.90) | 0.389 |
|  | **5-FU** | vs | **S-1** | 0.04 (0.00, 0.34) | 0.28 (0.01, 4.60) | 0.08 (0.01, 0.54) | 0.316 |
|  | **S-1+PAC** | vs | **S-1+IRI** | 0.23 (0.01, 3.60) | 5.90 (0.23, 510.00) | 0.89 (0.09, 5.90) | 0.134 |
|  | **S-1+CIS** | vs | **S-1+OXA** | 1.30 (0.27, 7.70) | 0.36 (0.01, 11.00) | 1.10 (0.25, 3.90) | 0.461 |
|  | **CAP+CIS** | vs | **S-1+CIS** | 0.45 (0.10, 1.70) | 0.66 (0.06, 4.30) | 0.53 (0.17, 1.50) | 0.731 |
|  | **5-FU+CIS** | vs | **S-1+CIS** | 0.63 (0.20, 1.40) | 0.27 (0.03, 2.40) | 0.57 (0.20, 1.10) | 0.429 |
|  | **5-FU+CIS** | vs | **CAP+CIS** | 0.87 (0.15, 5.40) | 1.30 (0.18, 6.10) | 1.10 (0.31, 3.20) | 0.741 |
|  | **5-FU** | vs | **5-FU+CIS** | 1.10 (0.02, 53.00) | 0.04 (0.00, 0.65) | 0.13 (0.01, 1.10) | 0.158 |
|  | **5-FU** | vs | **5-FU+IRI** | 0.05 (0.00, 0.61) | 0.01 (0.00, 0.32) | 0.03 (0.00, 0.27) | 0.509 |
| **Diarrhoea** | **S-1+IRI** | vs | **S-1** | 1.40 (0.47, 4.70) | 4.40 (0.15, 180.00) | 1.50 (0.56, 4.50) | 0.525 |
|  | **S-1+PAC** | vs | **S-1** | 1.80 (0.24, 14.00) | 0.47 (0.01, 11.00) | 1.30 (0.27, 6.50) | 0.463 |
|  | **S-1+OXA** | vs | **S-1** | 1.30 (0.21, 11.00) | 2.90 (0.25, 38.00) | 1.70 (0.44, 6.80) | 0.576 |
|  | **S-1+CIS** | vs | **S-1** | 3.50 (0.53, 42.00) | 1.70 (0.16, 19.00) | 2.40 (0.62, 12.00) | 0.638 |
|  | **S-1+PAC** | vs | **S-1+IRI** | 0.43 (0.01, 6.60) | 1.40 (0.16, 15.00) | 0.89 (0.14, 4.70) | 0.459 |
|  | **S-1+CIS** | vs | **S-1+OXA** | 1.30 (0.30, 4.50) | 3.10 (0.24, 55.00) | 1.50 (0.53, 4.90) | 0.523 |
|  | **CAP+CIS** | vs | **S-1+CIS** | 4.10 (0.96, 16.00) | 0.88 (0.02, 31.00) | 3.30 (0.96, 12.00) | 0.403 |
|  | **5-FU+CIS** | vs | **S-1+CIS** | 1.10 (0.43, 2.60) | 3.80 (0.06, 170.00) | 1.10 (0.55, 2.50) | 0.494 |
|  | **5-FU+CIS** | vs | **CAP+CIS** | 1.00 (0.01, 58.00) | 0.27 (0.05, 1.30) | 0.35 (0.09, 1.40) | 0.494 |
| **Fatigue** | **S-1+IRI** | vs | **S-1** | 1.40 (0.47, 4.70) | 4.40 (0.15, 180.00) | 1.50 (0.56, 4.50) | 0.525 |
|  | **S-1+PAC** | vs | **S-1** | 1.80 (0.24, 14.00) | 0.47 (0.01, 11.00) | 1.30 (0.27, 6.50) | 0.463 |
|  | **S-1+OXA** | vs | **S-1** | 1.30 (0.21, 11.00) | 2.90 (0.25, 38.00) | 1.70 (0.44, 6.80) | 0.576 |
|  | **S-1+CIS** | vs | **S-1** | 3.50 (0.53, 42.00) | 1.70 (0.16, 19.00) | 2.40 (0.62, 12.00) | 0.638 |
|  | **S-1+PAC** | vs | **S-1+IRI** | 0.43 (0.01, 6.60) | 1.40 (0.16, 15.00) | 0.89 (0.14, 4.70) | 0.459 |
|  | **S-1+CIS** | vs | **S-1+OXA** | 1.30 (0.30, 4.50) | 3.10 (0.24, 55.00) | 1.50 (0.53, 4.90) | 0.523 |
|  | **CAP+CIS** | vs | **S-1+CIS** | 4.10 (0.96, 16.00) | 0.88 (0.02, 31.00) | 3.30 (0.96, 12.00) | 0.403 |
|  | **5-FU+CIS** | vs | **S-1+CIS** | 1.10 (0.43, 2.60) | 3.80 (0.06, 170.00) | 1.10 (0.55, 2.50) | 0.494 |
|  | **5-FU+CIS** | vs | **CAP+CIS** | 1.00 (0.01, 58.00) | 0.27 (0.05, 1.30) | 0.35 (0.09, 1.40) | 0.494 |
| **Febrile Neutropenia** | **S-1+IRI** | vs | **S-1** | 2.00 (0.10, 43.00) | 0.94 (0.00, 210.00) | 2.00 (0.15, 23.00) | 0.775 |
|  | **S-1+PAC** | vs | **S-1** | 0.42 (0.01, 18.00) | 0.88 (0.00, 87.00) | 0.56 (0.04, 7.60) | 0.798 |
|  | **S-1+OXA** | vs | **S-1** | 5.50 (0.56, 62.00) | 0.24 (0.01, 4.60) | 1.80 (0.22, 18.00) | 0.084 |
|  | **S-1+CIS** | vs | **S-1** | 3.20 (0.16, 55.00) | 15.00 (0.63, 310.00) | 7.00 (0.73, 64.00) | 0.428 |
|  | **5-FU** | vs | **S-1** | 0.95 (0.02, 89.00) | 13.00 (0.27, 510.00) | 4.80 (0.15, 62.00) | 0.333 |
|  | **S-1+PAC** | vs | **S-1+IRI** | 0.38 (0.01, 15.00) | 0.17 (0.00, 19.00) | 0.28 (0.02, 5.60) | 0.805 |
|  | **S-1+CIS** | vs | **S-1+OXA** | 8.70 (1.10, 110.00) | 0.39 (0.01, 11.00) | 3.90 (0.41, 27.00) | 0.080 |
|  | **5-FU+CIS** | vs | **S-1+CIS** | 3.30 (0.34, 22.00) | 0.27 (0.00, 48.00) | 2.80 (0.22, 13.00) | 0.368 |
|  | **5-FU** | vs | **5-FU+CIS** | 0.40 (0.01, 12.00) | 0.04 (0.00, 14.00) | 0.26 (0.03, 2.20) | 0.429 |
|  | **5-FU** | vs | **5-FU+IRI** | 0.75 (0.03, 37.00) | 0.07 (0.00, 95.00) | 0.62 (0.05, 5.90) | 0.484 |
| **Leucopenia** | **S-1+IRI** | vs | **S-1** | 0.58 (0.09, 3.90) | 57.00 (0.97, 7400.00) | 1.30 (0.21, 10.00) | **0.034** |
|  | **S-1+PAC** | vs | **S-1** | 4.00 (0.23, 84.00) | 0.04 (0.00, 2.00) | 0.71 (0.06, 8.60) | 0.060 |
|  | **S-1+OXA** | vs | **S-1** | 2.20 (0.06, 64.00) | 1.60 (0.03, 98.00) | 2.00 (0.20, 21.00) | 0.890 |
|  | **S-1+CIS** | vs | **S-1** | 9.90 (0.74, 180.00) | 11.00 (0.07, 2400.00) | 10.00 (1.60, 76.00) | 0.978 |
|  | **5-FU+CIS** | vs | **S-1** | 12.00 (0.24, 1200.00) | 11.00 (0.33, 380.00) | 11.00 (1.20, 110.00) | 0.934 |
|  | **S-1+PAC** | vs | **S-1+IRI** | 0.08 (0.00, 1.60) | 6.30 (0.18, 210.00) | 0.55 (0.04, 6.00) | 0.063 |
|  | **S-1+CIS** | vs | **S-1+OXA** | 5.60 (0.22, 130.00) | 4.00 (0.05, 310.00) | 5.20 (0.55, 49.00) | 0.869 |
|  | **CAP+CIS** | vs | **S-1+CIS** | 0.53 (0.01, 26.00) | 0.68 (0.02, 23.00) | 0.60 (0.05, 6.90) | 0.878 |
|  | **5-FU+CIS** | vs | **S-1+CIS** | 1.10 (0.22, 6.10) | 0.76 (0.00, 110.00) | 1.10 (0.27, 4.10) | 0.884 |
|  | **5-FU+CIS** | vs | **CAP+CIS** | 1.60 (0.05, 38.00) | 2.50 (0.05, 220.00) | 1.80 (0.15, 22.00) | 0.834 |
| **Nausea** | **S-1+IRI** | vs | **S-1** | 0.85 (0.33, 2.10) | 5.00 (0.28, 72.00) | 1.00 (0.41, 2.50) | 0.225 |
|  | **S-1+PAC** | vs | **S-1** | 7.60 (1.40, 80.00) | 1.30 (0.15, 17.00) | 3.70 (1.10, 15.00) | 0.266 |
|  | **S-1+OXA** | vs | **S-1** | 1.60 (0.24, 20.00) | 11.00 (2.30, 74.00) | 5.20 (1.50, 23.00) | 0.183 |
|  | **S-1+CIS** | vs | **S-1** | 9.50 (2.80, 39.00) | 1.60 (0.15, 18.00) | 6.60 (2.40, 23.00) | 0.194 |
|  | **5-FU+CIS** | vs | **S-1** | 16.00 (2.30, 280.00) | 8.20 (2.20, 34.00) | 8.90 (3.20, 33.00) | 0.609 |
|  | **S-1+PAC** | vs | **S-1+IRI** | 1.50 (0.22, 13.00) | 8.50 (1.30, 120.00) | 3.70 (1.00, 16.00) | 0.193 |
|  | **S-1+CIS** | vs | **S-1+OXA** | 0.95 (0.35, 2.70) | 6.20 (0.53, 77.00) | 1.20 (0.50, 3.20) | 0.161 |
|  | **CAP+CIS** | vs | **S-1+CIS** | 0.91 (0.30, 2.80) | 0.99 (0.15, 5.30) | 0.91 (0.35, 2.40) | 0.931 |
|  | **5-FU+CIS** | vs | **S-1+CIS** | 1.40 (0.88, 2.20) | 1.20 (0.16, 13.00) | 1.30 (0.91, 2.00) | 0.919 |
|  | **5-FU+CIS** | vs | **CAP+CIS** | 1.40 (0.26, 9.10) | 1.50 (0.42, 5.30) | 1.50 (0.56, 4.10) | 0.965 |
| **Neutropenia** | **S-1+OXA** | vs | **S-1** | 2.90 (0.30, 34.00) | 1.70 (0.14, 20.00) | 2.30 (0.45, 12.00) | 0.735 |
|  | **S-1+CIS** | vs | **S-1** | 5.80 (1.00, 32.00) | 4.90 (0.43, 50.00) | 5.10 (1.70, 19.00) | 0.923 |
|  | **5-FU+CIS** | vs | **S-1** | 5.20 (0.49, 78.00) | 7.40 (1.20, 47.00) | 6.70 (1.80, 25.00) | 0.849 |
|  | **5-FU** | vs | **S-1** | 0.19 (0.02, 2.00) | 0.52 (0.03, 5.90) | 0.30 (0.05, 1.60) | 0.564 |
|  | **S-1+CIS** | vs | **S-1+OXA** | 2.90 (0.38, 21.00) | 1.50 (0.10, 19.00) | 2.20 (0.48, 10.00) | 0.666 |
|  | **CAP+CIS** | vs | **S-1+CIS** | 1.10 (0.24, 4.60) | 1.00 (0.09, 9.50) | 1.10 (0.33, 3.40) | 0.955 |
|  | **5-FU+CIS** | vs | **S-1+CIS** | 1.40 (0.45, 4.00) | 0.93 (0.11, 8.20) | 1.30 (0.50, 3.20) | 0.701 |
|  | **5-FU+CIS** | vs | **CAP+CIS** | 1.20 (0.14, 11.00) | 1.10 (0.20, 7.50) | 1.20 (0.30, 4.50) | 0.961 |
|  | **5-FU** | vs | **5-FU+CIS** | 0.07 (0.01, 0.77) | 0.02 (0.00, 0.45) | 0.05 (0.01, 0.23) | 0.523 |
|  | **5-FU** | vs | **5-FU+IRI** | 0.18 (0.02, 2.10) | 0.09 (0.00, 3.30) | 0.15 (0.02, 0.88) | 0.728 |
| **Stomatitis** | **S-1+IRI** | vs | **S-1** | 0.85 (0.09, 9.60) | 6.70 (0.05, 2300.00) | 1.10 (0.16, 10.00) | 0.449 |
|  | **S-1+PAC** | vs | **S-1** | 2.50 (0.07, 170.00) | 0.32 (0.00, 21.00) | 1.20 (0.07, 19.00) | 0.469 |
|  | **S-1+OXA** | vs | **S-1** | 1.00 (0.01, 85.00) | 1.40 (0.02, 94.00) | 1.10 (0.08, 26.00) | 0.906 |
|  | **S-1+CIS** | vs | **S-1** | 2.50 (0.06, 190.00) | 0.46 (0.02, 12.00) | 0.98 (0.09, 13.00) | 0.484 |
|  | **5-FU** | vs | **S-1** | 1.90 (0.12, 44.00) | 8.70 (0.05, 990.00) | 3.10 (0.34, 44.00) | 0.601 |
|  | **S-1+PAC** | vs | **S-1+IRI** | 0.40 (0.01, 13.00) | 3.00 (0.04, 400.00) | 0.93 (0.06, 20.00) | 0.450 |
|  | **S-1+CIS** | vs | **S-1+OXA** | 0.79 (0.05, 19.00) | 1.10 (0.01, 240.00) | 0.81 (0.07, 10.00) | 0.885 |
|  | **CAP+CIS** | vs | **S-1+CIS** | 1.00 (0.07, 15.00) | 1.30 (0.02, 38.00) | 1.10 (0.13, 8.00) | 0.913 |
|  | **5-FU+CIS** | vs | **S-1+CIS** | 7.30 (0.46, 51.00) | 2.50 (0.09, 59.00) | 5.20 (0.66, 27.00) | 0.584 |
|  | **5-FU+CIS** | vs | **CAP+CIS** | 3.90 (0.17, 81.00) | 5.50 (0.14, 130.00) | 4.80 (0.53, 36.00) | 0.869 |
|  | **5-FU** | vs | **5-FU+CIS** | 4.00 (0.21, 190.00) | 0.17 (0.00, 13.00) | 0.62 (0.06, 8.20) | 0.203 |
|  | **5-FU** | vs | **5-FU+IRI** | 1.00 (0.07, 16.00) | 1.70 (0.03, 210.00) | 1.90 (0.15, 25.00) | 0.814 |
| **Thrombocytopenia** | **S-1+OXA** | vs | **S-1** | 6.20 (0.39, 110.00) | 9.20 (0.21, 770.00) | 6.70 (0.78, 69.00) | 0.864 |
|  | **S-1+CIS** | vs | **S-1** | 9.70 (0.38, 420.00) | 6.70 (0.23, 300.00) | 7.40 (0.74, 100.00) | 0.846 |
|  | **S-1+CIS** | vs | **S-1+OXA** | 1.00 (0.10, 11.00) | 1.40 (0.02, 240.00) | 1.10 (0.17, 7.90) | 0.864 |
| **Vomiting** | **S-1+OXA** | vs | **S-1** | 2.10 (0.15, 58.00) | 1.20 (0.12, 11.00) | 1.70 (0.32, 9.70) | 0.710 |
|  | **S-1+CIS** | vs | **S-1** | 3.20 (1.00, 14.00) | 8.60 (0.25, 360.00) | 3.90 (1.30, 15.00) | 0.670 |
|  | **5-FU+CIS** | vs | **S-1** | 18.00 (2.30, 310.00) | 3.50 (0.89, 18.00) | 5.20 (1.70, 21.00) | 0.245 |
|  | **S-1+CIS** | vs | **S-1+OXA** | 2.70 (0.48, 22.00) | 1.70 (0.04, 32.00) | 2.40 (0.49, 11.00) | 0.789 |
|  | **CAP+CIS** | vs | **S-1+CIS** | 1.10 (0.16, 7.50) | 1.10 (0.36, 3.40) | 1.10 (0.45, 2.90) | 0.968 |
|  | **5-FU+CIS** | vs | **S-1+CIS** | 1.30 (0.83, 2.10) | 1.30 (0.18, 11.00) | 1.30 (0.88, 2.10) | 0.966 |
|  | **5-FU+CIS** | vs | **CAP+CIS** | 1.20 (0.44, 3.60) | 1.30 (0.18, 8.10) | 1.20 (0.49, 3.10) | 0.983 |

Note: the **bold** form points out inconsistency.
